# Nutrients Lowering Obesity-Linked Chemokines Blamable for Metastasis

**DOI:** 10.3390/ijms26052275

**Published:** 2025-03-04

**Authors:** Gabriela Ion, Marinela Bostan, Wanda Elaine Hardman, Margaret Putt McFarland, Coralia Bleotu, Nicoleta Radu, Carmen Cristina Diaconu, Mirela Mihaila, Mihai Dan Caramihai, Camelia Mia Hotnog

**Affiliations:** 1 Center of Immunology, Stefan S. Nicolau Institute of Virology, Romanian Academy, 030304 Bucharest, Romania; gabriela.ion@virology.ro (G.I.); camelia.hotnog@virology.ro (C.M.H.); 2 Department of Immunology, ‘Victor Babes’ National Institute of Pathology, 050096 Bucharest, Romania; 3 Department of Biomedical Sciences, Joan C. Edwards School of Medicine, Marshall University, Huntington, WV 25701, USAputt@marshall.edu (M.P.M.); 4 Department of Cellular and Molecular Pathology, Stefan S. Nicolau Institute of Virology, Romanian Academy, 030304 Bucharest, Romania; coralia.bleotu@virology.ro (C.B.); carmen.diaconu@virology.ro (C.C.D.); 5 Research Institute of the University of Bucharest (ICUB), University of Bucharest, 060023 Bucharest, Romania; 6 The Academy of Romanian Scientist, 050711 Bucharest, Romania; 7 Faculty of Biotechnology, University of Agronomic Sciences and Veterinary Medicine of Bucharest, 011464 Bucharest, Romania; nicoleta.radu@biotehnologii.usamv.ro; 8 Biotechnology Department, National Institute for Chemistry and Petrochemistry R&D of Bucharest, 060021 Bucharest, Romania; 9 Faculty of Pharmacy, Titu Maiorescu University, 040314 Bucharest, Romania; 10 Faculty of Automatic Control and Computer Science, National University of Science and Technology Politehnica Bucharest, 060042 Bucharest, Romania; m.caramihai@yahoo.com; 11 Department of Biochemistry and Biophysics, Faculty of Midwives and Nursing, University of Medicine and Pharmacy “Carol Davila” Bucharest, 050474 Bucharest, Romania

**Keywords:** nutrients, chemokines, obesity, metastasis

## Abstract

Food intake is an essential contributor to both health and disease. Nutrients contribute to a beneficial metabolic equilibrium at the cellular level, preventing or delaying disease onset. Dietary intake contributes to obesity, and obesity supports further cancer and metastasis. Metastasis, a multifactorial and multistep process, is supported by the systemic inflammation of obesity. Spreading of the cancer cells requires the presence of a plethora of recruiter and regulator molecules. Molecules such as chemokines are provided at high levels by obesity-associated fat depots. Chemokine up-regulation in adipose tissue of obese individuals has been associated with different types of cancers such as breast, prostate, colon, liver, and stomach. Chemokines support all metastasis steps from invasion/migration to intravasation, circulation, extravasation, and ending with colonization. The obesity pool of chemokines supporting these processes includes CCL2, CCL3, CCL4, CCL5, CCL7, CCL8, CCL11, CCL18, CCL19, CCL20, CXCL1, CXCL5, CXCL 8, CXCL10, and CXCL12. Keeping obesity under control can be beneficial in reducing the levels of pro-inflammatory chemokines and the risk of poor cancer outcome. Nutrients can help, support, and boost cancer treatment effects or jeopardize the treatment. Constituents with anti-inflammatory and anti-obesity properties such as polyphenols, organosulfur components, fatty acids, curcumin, and vitamin E have a proven beneficial effect in lowering obesity and its contribution to metastasis.

## 1. Introduction

Obesity is a well-recognized risk factor for cancer development. The Centers for Disease Control and Prevention (CDC) listed 13 types of cancer associated with obesity [1]. Besides the growing evidence showing the positive impact of obesity metabolic disturbance on tumor development [2], emerging data support the chronic inflammation induced by obesity as an add-on to the negative effect of obesity on human health [3]. This review summarizes and synthesizes chemokines associated with obesity-related inflammation, cancer, and metastasis and their modulation by nutrients able to reduce the detrimental effect of excess adiposity.

**Adipose tissue cells** excrete soluble molecules such as cytokines, chemokines, adipokines, growth factors, and proteases that act on the neighboring cells to maintain the body’s physiological equilibrium. An imbalance in the signaling network can lead to and sustain inflammation. Thus, obesity sustaining inflammation is an important player in tumor development.

Epidemiological studies support the contribution of obesity to the increased rate of mortality in different cancers (colon, breast, esophagus, pancreas, kidney, and liver) [4]. Under physiological conditions, the role of inflammation is to restore homeostasis. However, an excessive inflammatory response has harmful effects. As a consequence, studies are showing that obesity can be responsible for kinase activation in the liver tissue, such as c-jun N-terminal kinase (JNK), which can be in charge of a high cytokine production [5]. High levels of circulating pro-inflammatory cytokines are associated not only with obesity per se but also with different associated diseases, including cancer [6]. Besides cytokines, obesity systemic inflammation provides a pool of chemokines favoring and supporting metastasis. Inflammation, as a deep-rooted path of obesity’s contribution to cancer progression [7,8], follows chemokines and chemokine receptor activation [9].

Chemokines produced by adipose tissue with a role in metastasis are chemokine (C-C motif) ligand (CCL) CCL2, CCL3, CCL4, CCL5, CCL7, CCL8, CCL11, CCL18, CCL19, CCL20, C-X-C motif chemokine ligand (CXCL) CXCL1, CXCL5, CXCL 8, CXCL10, CXCL12, and their receptors C-C chemokine receptor type (CCR) CCR1, CCR2, CCR3, and CCR5. Among the chemokines involved in all steps of the metastatic process (invasion/migration, intravasation, circulation, extravasation, and colonization) a very well established and depicted axis is CCL2/CCR2 [10] (Table 1).

CCL3 is another well-described chemokine involved in all metastasis steps (Table 1) by making pairs either with CCR5 [25] or CCR1 [26]. CCL4 involvement in cancer development has a long history. In spite of the fact that its contribution to cancer development was first mentioned in 1964 [27,28], like all other chemokines, the data linking CCL4 and metastasis only emerged in the last 2–3 decades.

In addition to excess food consumption, **the quality of the food** can make a difference between health and disease. Fruits, vegetables, and cooked food can improve obesity’s negative impact and decrease the inflammation interconnected with it. **Organosulfur components, healthy fats, vitamin E, and polyphenols** (curcumin, flavonoids, resveratrol, and stilbenes) have beneficial effects by reducing obesity, inflammation, and associated diseases, including the spread of cancer cells (Table 2). This effect is supported by a tremendous amount of literature data. Thus, organosulfur components lower adipogenesis, adipose tissue inflammation, cancer development, and CCL2 and CXCL12 release [29,30,31,32,33,34,35,36,37,38].

Vitamin E lowers CCL2 [39,40]. Curcumin is well recognized as decreasing adipogenesis, adipose tissue inflammation, and cytokine and chemokine (CCL2, CCL5, CCL7, CXCL1, CXCL2, CXCL10, CXCL12, and CXCL12/CXCR4) release [41,42,43,44,45,46,47,48,49,50,51,52,53,54,55,56,57,59,60,93,94]. Similarly, flavonoids (epicatechin, epigallocatechin-3-gallate, naringenin, genistein, quercetin, flavonoid fish-oil supplement, anthocyanins, baicalin, cirsimarin, and apigenin) lower adipogenesis, adipose tissue inflammation, and chemokine (CCL2, CCL5, CCL8, CCL19, CXCL8, CXCL12, and CXCR4) release [61,62,63,64,65,66,67,68,69,70,71,72,73,74,77,78,79].

**Omega-3 polyunsaturated fatty acids (n-3 FAs)** are important players in both obesity and cancer prevention. A well-balanced n-3–n-6 ratio is essential for good human health [95]. N-3 FAs have well-known anti-inflammatory properties [96]. A high n-3 diet decreases prostate [97], mammary [98,99,100], lung [101], and colorectal [102,103] carcinogenesis. N-3 FAs are present in foods such as fish (salmon, mackerel), nuts (walnuts), seeds (flaxseed) and their oils, and other plant oils (canola, soybean). Among n-3 FAs, α-linolenic acid (ALA), eicosapentaenoic acid (EPA), and docosahexaenoic acid (DHA) are the most used for human health. Since ALA is an essential fatty acid and cannot be synthetized by the body, it must be provided by food. The human body can synthetize EPA and DHA from their precursor, ALA, at a low rate. Therefore, a healthy status is supported by foods and supplements rich in ALA, EPA, and DHA.

Since obesity has become an epidemic, adipose tissue is now under extensive study, and new insights are being revealed regarding the complexity of its functionality. When healthy adipose tissue has a good balance of secreted products (e.g., chemokines), it can make a difference in the outcome of different diseases including cancer. Mechanisms involved in inflammatory processes induced by obesity that lead to tumor development need a more thorough understanding. In this regard, two points are pertinent: (i) how we can lower the inflammation induced by obesity in order to prevent or slow tumor progression and/or resistance to the classic chemotherapeutics; and (ii) how food intake can reduce the excess of adipose tissue, inflammation, and obesity-induced chemokine levels in the hope of lowering the risk of obesity-favored tumors.

## 2. Adipose Tissue in Obesity

Body fat is a widely spread organ with multiple functions (energy storage, thermogenesis, secretion of hormones, adipokines, cytokines, and chemokines). A simple classification of body fat is by morphology (white, brown, and beige) [104]. The most common classification is by localization (e.g., upper body, lower body, subcutaneous, visceral, and ectopic) [104,105,106,107]. Accumulation and enlargement of normally distributed adipose tissue and the ectopic localization of the fat depots are characteristic for obesity. The ectopic localization of adipose tissue affects tissues and organs such as the liver, muscles, vasculature, epicardium, and kidneys. The main cell type presented in adipose tissue is adipocytes. In addition, several other cell types are part of the tissue: preadipocytes, stromal/stem cells, fibroblasts, macrophages, and endothelial cells. Chronic inflammation benefits from the support of a widely distributed organ composed of different cell types that secrete a plethora of pro-inflammatory molecules.

Obesity-associated body fat influences not only the release of pro-inflammatory molecules but also directly interacts with tumor cells influencing their behavior. As a response to the changes in the microenvironment caused by obesity, tumor cells adjust the expression of chemokine receptors (such as CXCR4, CXCR7, and CCR2). This adjustment allows them to migrate to tissues (such as distant organs) where chemokines are elevated. Thus, tumor cells are intrinsically able to support both adipose tissue and tumor microenvironment inflammation. This leads to the recruitment of inflammatory cells generating a positive feedback loop that further support tumor progression and metastasis [108].

## 3. Chemokine Contribution to Obesity Chronic Inflammation and Metastasis

Chemokines (chemotactic cytokines) initially established as leukocyte recruiters [109] play a key role in tumor cell trafficking and metastasis [110]. In this context, a particular interest in the obesity systemic inflammation might focus on perivascular adipose tissue. The recent focus has mainly been on studies showing the role of perivascular adipose tissue cytokines/chemokines in the health of vasculature. A study published by Chatterjee TK et al. in 2009 supports the pro-inflammatory action of a high-fat diet on perivascular adipocytes. The authors showed that **CCL2** (alias monocyte chemoattractant protein 1 (MCP-1)) is increased in perivascular adipocytes due to diet. Moreover, the amount of CCL2 is depot specific [11]. CCL2 as a pro-inflammatory molecule is an essential recruiter of macrophages to both adipose tissue and tumor microenvironment. Another study showed that, besides CCL2, the perivascular adipose tissue secretes **CXCL8** (alias interleukin 8, IL-8) [12]. CXCL8 has a well-defined role in metastasis by facilitating tumor cell migration, increased vascular permeability, and new blood vessel formation [111]. Thus, high CXCL8 levels are associated with the presence of different cancers (oesophageal, gastric, pancreatic, breast, and kidney cancer) [112,113,114,115,116], metastasis, and drug resistance [116]. In order to support both metastasis and drug resistance, CXCL8 activates the serine/threonine kinase Akt pathway (protein kinase B) via CXCR2 [116].

The expression of CXCL8 alongside the chemokines CCL2, CCL5, CCL7, CCL19, CXCL1, CXCL5, and CXCL10 was found to be up-regulated in the subcutaneous adipose tissue of obese subjects [14] (Table 1). Moreover, the gene expression of CCL2, CCL3, CCL5, CCL7, CCL8, CCL11, and their receptors (CCR1, CCR2, CCR3, and CCR5) was found to be higher in both the subcutaneous and visceral adipose tissue of obese individuals [9].

**CCL3** (alias macrophage inflammatory proteins-1α (MIP-1α)) and **CCL4** (MIP-1β), secreted by macrophages and T cells and also by adipocytes [9,15,17], contribute to chronic inflammation by recruiting monocytes, T cells, and neutrophils to the inflammatory sites. In the tumor microenvironment, CCL3 and CCL4 help to establish a favorable niche for cancer cell dissemination.

**CCL5** (alias regulated on activation normal T-cell expressed and secreted (RANTES)) associated with obesity is an important recruiter of monocytic myeloid-derived suppressor cells (MDSCs). Under obese conditions, CCL5/CCR5 signaling contributes to adipose tissue inflammation by enabling MDSC differentiation to tissue macrophages [117]. Moreover, CCL5 facilitates the migration and invasion of tumor cells through its interaction with CCR5 receptors present on these cells. Elevated concentrations of CCL5 within the tumor microenvironment contribute to immune evasion by recruiting immune cells that promote the survival and migration of cancer cells, thereby heightening the risk of metastasis [118,119,120,121].

**CXCL1** (alias growth-regulated protein alpha Gro alpha, Gro1, melanoma growth-stimulating activity, alpha (MGSA-α)) released by adipocytes and preadipocytes [13] is associated with invasive breast cancer and metastasis. The process is supported by CXCL1 recruiting myeloid cells to tumors [122] through the nuclear factor kappa-light-chain-enhancer of activated B cells (NF-κB)/SRY (sex-determining region Y)-Box Transcription Factor 4 (SOX4) activation [123]. CXCL1 is a strategic player in bladder cancer, supporting repeated intravesical recurrence and disease progression [124].

**CXCL12** (alias stromal cell-derived factor 1, SDF-1) is defined as a macrophage chemotactic factor released by adipocytes, stromal cells, and various cancer cells. It is a potent attractant for different cell types (immune, endothelial, and tumor cells), expressing its receptor, CXCR4. And its increased levels are observed in adipose tissue and tumors associated with obesity [23]. CXCL12 and its receptor CXCR4 are associated with the development of both different organ tumors and metastasis. Therefore, CXCL12 not only helps cancer cells find the nest, but also improves tumor-associated inflammation, facilitating the establishment of a metastatic niche [125,126,127].

Adipose tissue contributes not only to a local increase in chemokines, it can also contribute to a rise in serum chemokine levels. In this respect, high serum levels of chemokines have been found in different types of cancers. CCL2 is high in both serum and tumor tissue from patients with non-small-cell lung cancer [128]. Serum levels of CCL2 are also high in patients with ovarian cancer [129], nasopharyngeal carcinoma [130], and pancreatic cancer compared to healthy subjects [131]. CCL3 has been found to be high in the plasma of patients with non-small-cell lung carcinoma [132]. CCL4, CCL5, and CXCL5 are high in the serum of patients with hepatocellular carcinoma [133]. Human epidermal growth factor receptor 2 (Her2) (alias receptor tyrosine-protein kinase erbB-2 (ERBB2) or cluster of differentiation 340 (CD340))-positive breast cancer patients have high serum levels of CCL5 associated with poor prognosis [134]. CXCL8 has been found to be higher in the serum of patients with oesophageal, gastric, pancreatic, breast, or kidney cancer [112,113,114,115,116]. A high serum expression of CXCL8 is correlated with poor outcome of disease [116]. High plasma CXCL10 levels among other factors are associated with a poor outcome in metastatic renal cell carcinoma patients treated with antiangiogenic therapy [135]. CCL11 is elevated in the serum of patients with esophageal squamous cell carcinoma [136] or gastric cancer [137]. High serum CCL18 is associated with a poor prognosis in patients with different carcinoma such as laryngeal squamous cell [138], squamous cell carcinoma of the head and neck [139], breast cancer [140], non-small-cell lung carcinoma [141], and pancreatic ductal carcinoma [142].

## 4. Management of Obesity and Associated Tumor Processes

**Figure 1:**
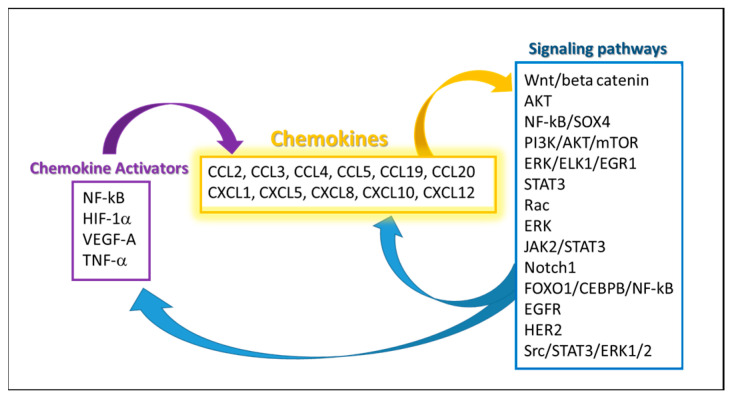
Chemokines released by adipose tissue are part of multiple signaling loops that support inflammation, cancer, metastasis, and drug resistance.

Adipose tissue in obesity provides a microenvironment characterized by endo/para and autocrine changes that may promote the initiation and/or progression of tumor processes. In this context, adipose tissue metabolic processes drive the long-term pro-inflammatory status. Among the processes supporting chronic inflammation is hypoxia. The partial pressure of oxygen in the adipose tissue of obese individuals is lower than in lean individuals [143]. Hypoxia occurring in the fat tissue of obese individuals is responsible for the increased expression of transcription factors: NF-κB and hypoxia-inducible factor-1α (HIF-1α). Both chronic and cyclic hypoxia maintain a high pro-inflammatory status [144]. Some studies emphasize that cyclic hypoxia might induce a higher expression of NF-κB and a greater pro-inflammatory response than chronic hypoxia [145]. Such a response triggers the release of CCL2, CXCL1, and CXCL8 [145] (Figure 1). CCL2 released by omental adipocytes facilitates the migration and omental metastasis of ovarian cancer through the activation of PI3K/AKT/mTOR followed by an increase in HIF-1α and vascular endothelial growth factor A (VEGF-A) [146]. The release of HIF-1α influences the synthesis of a large variety of proteins, such as collagen, metalloproteinase, metalloproteinases inhibitors, cytokines (interleukin 6 (IL-6), TNF), chemokines (CCL2, CXCL8, and CXCL12), and chemokine receptors (CXCR4) contributing to cell proliferation and metastasis [127,147,148,149,150,151]. High HIF-1α and CXCL8 are associated with the development of hepatocellular carcinoma and metastasis [151]. HIF also induces high CXCL12/CXCR4 responsible for breast cancer progression and metastasis [127]. CXCL12 is defined as a hypoxia-regulated gene strongly linked to carcinogenesis being responsible for drug resistance and metastasis [144].

Another process contributing to obesity systemic inflammation supporting metastasis is aerobic glycolysis. This process generates nutrients (lactate and pyruvate) required by tumor cells to complete mitochondrial oxidative metabolism. The process is known as the inverted Warburg effect, in which the mitochondria of tumor cells, via the process of beta-oxidation, use free fatty acids generated through lipolysis by stromal adipocytes [152,153]. Aerobic glycolysis is supported by the infiltration of macrophages into the white adipose tissue. Once activated, so-called M1 macrophages are pro-inflammatory and induce aerobic glycolysis [154]. Among the stimuli that activate M1 macrophages, there are CCL8, 19, 20, and CXCL10 [154]. These stimuli are released either by preadipocytes or adipocytes [13]. Glycolysis also requires the presence of hypoxia-activated glycolytic genes (aldolase, fructose-bisphosphate C (ALDOC), enolase 2 (ENO2), hexokinase (HK1, 2), and phosphofructokinase, platelet (PFKP)) [143,144].

NF-κB activation is present in animals fed with a high-fat diet, as well as in obese individuals [155,156,157]. It is responsible for maintaining an inflammatory environment by triggering chemokines CCL 3, 4, 5, 19, 20 and CXCL 1, 5, 8, 10 [158,159] (Figure 1), the survival of adipose tissue macrophages [160], and a positive feedback loop CXCL 1, 8 -NF-κB [159]. Moreover, NF-κB supports white adipose tissue (WAT) inflammation throughout cachexia. NF-κBp65 and its target CCL2 were found to be higher in adipose tissue of cancer cachectic patients compared to non-cachectic cancer or cancer-free patients [161].

Another contributor to tumor development and metastasis delivered by adipose tissue is adipose stromal/stem cells (ASCs). ASCs, largely present in WAT, have an obesity-associated high proliferative capability and can migrate from WAT to the tumor site, contributing to tumor progression [162,163,164]. The trafficking of ASCs is supported by chemokines that have a role in invasion/migration and colonization such as CXCL1 and CXCL8 [165,166]. There are studies showing that ASCs are able to differentiate into carcinoma-associated fibroblasts supporting tumor invasion [167]. The ASC tumor-promoting effect is documented for different cancers: breast [168], ovarian [22], and prostate [24]. The metastasis of breast cancer xenografts is induced by human ASCs [169]. Prostate cancer chemoresistance is associated with ASCs releasing CXCL12 [24]. Also, ASCs have a high expression of CCR5 and release CXCL-10 in ovarian cancer [22].

In a study conducted by Arendt LM, a so-called adipose stromal vascular fraction, comprising adipocytes (stem cells, pre- and mature adipocytes) and other stromal cells, recruits macrophages through CCL2/CXCL12 signaling [170]. The recruitment of macrophages, as was mentioned above, is a pillar in sustained inflammation and tumor progression supported by obesity.

In addition to ASCs, another set of interactions involves cancer-associated adipocytes (CAAs) as an interface between adipocytes and tumor cells. Compared to adipocytes, CAAs have a smaller size and lipid droplets, and a different pattern of differentiation markers [171]. To transform into CAAs, adipocytes need to go through lipolysis, which is induced by tumor cells [171]. CAAs are better equipped to support tumor invasion and metastasis by releasing CCL2, CCL5, CXCL8, IL-6, leptin, adiponectin, and VEGF [171,172]. CAAs are defined as high producers of CXCL8 [173], CCL2, and IL-6 [174] associated with breast cancer development. Also, the release of CCL2, CXCL1, and Il-6 by CAAs is associated with bladder cancer cell migration [175].

## 5. Adipose Tissue, Chemokines, and Chemo-Resistance

Chemoresistance benefits from the support of adipose tissue environment. Therefore, adipocytes are responsible for the resistance of acute lymphoblastic leukemia cells to daunorubicin and vincristine. One possible mechanism of daunorubicin resistance is the oxidative stress protection provided by adipocytes to leukemia cells [176]. Resistance to vincristine might be induced by adipocytes concealing the drug and increasing the expression of survival signaling [177]. Literature covering the support of adipose tissue in breast cancer development is very well documented. Studies are showing that adipocytes and adipose-derived stem cells are responsible for breast cancer cell resistance to tamoxifen and paclitaxel, respectively [178,179].

**CCL2** has a proven role in drug resistance by activating the PI3K-Akt-mTOR signaling pathway in different cancers: breast [180], gastric [181], glioma [182], lung [183], and ovarian [184]. In order to exert its role in drug resistance, CCL2 is a part of different feedback loops such asNF-κB [181,184]; extracellular signal-regulated kinase (ERK)-ETS domain transcription factor 1 (ELK1)-early Growth Response 1 (EGR1) [185] (Figure 1).

**CCL5** signaling through beta-catenin/signal transducers and activators of transcription 3 (STAT3) promotes both metastasis and drug resistance in prostate cancer [119,120]. A 2013 study on prostate cancer patients showed that the CCL5 serum levels were not different among patients groups: (i) negative prostate biopsy, (ii) initial diagnosis of prostate cancer, and (iii) taxane-resistant groups [186]. The study emphasizes the importance of CCR1 in the development of taxane-resistant prostate cancer. In a cell culture setup, the CCR1/CCL5 interaction supports the invasion of taxane-resistant prostate cancer cells through the activation of ERK and Rac signaling pathways [186]. Also, CCL5 signaling is associated with glioblastoma resistance to temozolomide [187]. CCL5 decreases breast cancer cell responsiveness to epirubicin. Moreover, CCL5 enhances breast cancer migration and invasion accompanied by increased vimentin and decreased e-cadherin expression [121]. High CCL5 expression has a role in breast cancer cell resistance to trastuzumab by ERK phosphorylation [134] and tamoxifen by STAT3 activation [188]. Platinum-based therapy-resistant ovarian cancer patients have a higher expression of CCL5 compared to chemo-sensitive patients [189]. High CCL5 expression promotes chemo-resistance through the STAT3 and PI3K/AKT signaling pathways [189]. Additionally, CCL5 supports the aggressiveness of ovarian cancer by attracting regulatory T cells [118]. Up-regulation of CCL5 is also present in bevacizumab-resistant head and neck squamous cell carcinoma [190].

**CCL18** is associated with the chemo-resistance of lung cancer cells to cisplatin [191]. CCL20 has a role in crizotinib resistance in non-small-cell lung cancer. This drug resistance is induced by the activation of angiogenesis through Janus kinase 2 (JAK2)/STAT3-CCL20-VEGFA/IL6 [192]. High expression of CCL20 suppresses the response to immunotherapy and is linked to epithelial–mesenchymal transition and the TNF signal pathway in lung adenocarcinoma [193]. Similarly, in pancreatic ductal adenocarcinoma, CCL20 is associated with NF-κB-mediated TNF-related apoptosis-inducing ligand (TRAIL) resistance [194]. In ovarian cancer, it is associated with the resistance to paclitaxel via the Notch1 pathway [195] and the regulation of ABCB1 expression [196]. In chemo-resistant colorectal cancer, CCL20 recruits Tregs to support the process through the forkhead box protein O1 (FOXO1)/CCAAT/enhancer-binding protein beta (CEBPB)/NF-κB pathway [197] (Figure 1).

**CXCL1** supports breast, bladder, and colorectal cancer cell survival under chemotherapy. A study published by Acharyya S. et al. in 2012 showed that breast cancer has high expression of CCL20 and CXCL3 as a response to drugs. The response to chemotherapy elicits the TNF-α-CXCL1/2-S100A8/9 paracrine pathway, leading to chemo-resistance and metastasis [122]. In colorectal cancer, a high CXCL1/5 expression is maintained via the CXCR/MMPI/epidermal growth factor (EGF) pathway [198]. Moreover, colon cancer patients under cetuximab therapy have the serum levels of CXCL1/5 correlated with the presence of Ras/Raf mutation [198].

**CXCL5** high expression in bladder cancer is associated with mitomycin C resistance via NF-κB activation [199], and in kidney and breast cancer, it is associated with resistance to lysosomotropic drugs (sunitinib, lapatinib, and chloroquine) [200]. In lung cancer, CXCL5 induces resistance to checkpoint inhibitors [201].

**CXCL8** is well recognized for its contribution to chemo-resistance and activation of pro-survival pathways. CXCL8 is associated with resistance to multiple chemotherapeutic approaches including platinum-based drugs in ovarian cancer [202], epidermal growth factor receptor (EGFR) inhibitors in lung cancer [203], cisplatin and doxorubicin in hepatocellular carcinoma [204], and gemcitabine in pancreatic cancer [205]. Pancreatic cells respond to gemcitabine treatment by a boosted expression of CXCL8 following ROS generation and NF-κB activation [206]. Similarly, melanoma cells respond to dacarbazine by a high level of CXCL8 in a NF-κB-dependent manner [207]. High expression of CXCL8 in colorectal cancer is part of the Myc/CXCL8/tissue inhibitor of metalloproteinase 1 (TIMP1) oncogenic mark [208]. Thus, it regulates resistance to cetuximab through the activation of the EGFR pathway [209] and doxorubicin by the modulation of multidrug resistance 1 (MDR1) via inhibitor of nuclear factor kappa-B kinase subunit beta 1 (IKK-β/p65) [210]. PI3K, Her2, JAK, and CXCL8 network signaling is responsible for the resistance to PI3K inhibitors [211]. The CXCL8 drug resistance system is supported by recent studies showing connector molecules like Src homology-2 domain-containing protein tyrosine phosphatase-2 (SHP2), which mediates the CXCL8-CXCR1/2 feedback loop through ERK-AKT-NFκB and Src homology-2 domain-containing protein tyrosine phosphatase-2 (GSK3β)-β-catenin signaling [212]. Her-2-positive breast cancer resistance to lapatinib is induced by CXCL8 through the activation of Src/STAT3/ERK1/2-mediated EGFR signaling [213]. CXCL8 exerts its action by triggering multidrug resistance genes like ABCB1 in tumor blood vessels [214], ABCB5 in mesothelioma [215], and ABCB 1 in gastric cancer [216] and increasing the expression of NF-κB-regulated antiapoptotic genes like B-cell lymphoma 2 (Bcl-2) and inhibitor of apoptosis (IAP) families [217].

**CXCL10** is associated with breast cancer resistance to tamoxifen [218], pancreatic cancer resistance to gemcitabine [219], renal carcinoma resistance to sunitinib and pazopanib [135], and childhood acute lymphoblastic leukemia resistance to chemotherapy-induced apoptosis [220].

**CXCL12** has a well-established role in gastrointestinal cancer resistance to chemotherapy, where it is linked to disease progression, anti-programmed cell death protein 1 (anti-PD-1) [221], and 5-fluorouracil resistance [222]. In pancreatic cancer, it is associated with gemcitabine resistance through the activation of FAK, ERK, AKT, β-catenin, and NF-κB [223] and the induction of special AT-rich sequence-binding protein-1 (SATB-1) [224]. Moreover, it is associated with ovarian cancer resistance to cisplatin via the Wnt/β-catenin pathway [225]. CXCL12 plays a role in the resistance of leukemia cells to tyrosine kinase inhibitors [226]. In colorectal cancer, CXCL12 mediates resistance to 5-fluorouracil by targeting miR-125b [227]. Glioblastoma cell resistance to temozolomide is mediated by CXCL12 by forkhead box protein M1 (FOXM1) up-regulation [228]. High expression of CXCL12 is linked with MDR1 overexpression in chronic myelogenous leukemia [229].

## 6. Nutrients Disabling the Detrimental Effect of Excess Adiposity

**Figure 2:**
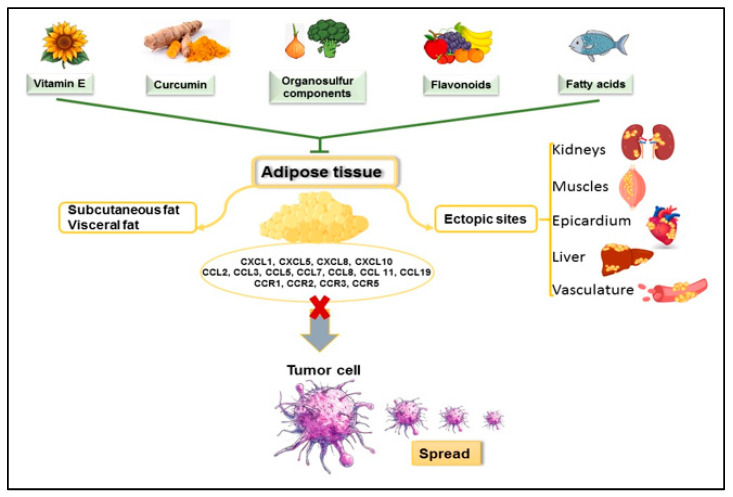
The spread of tumor cells supported by obesity can be impaired by healthy foods. Through their beneficial effect, nutrients can reduce the release of pro-inflammatory chemokines from adipose tissue.

Nutrients can act as a support for body health or as a disease trigger. Components with excellent anti-inflammatory and antioxidant properties can lower obesity-related chemokine levels (Figure 2).

### 6.1. Vitamin E

CCL2 seems to be an essential target of vitamin E since CCL2 levels greatly decrease in individuals taking vitamin E supplements [39]. Vitamin E family members, such as tocotrienols, improve the adverse effect of TNF-α in adipocytes by lowering NF-κB activation and CCL2 secretion [40]. Besides supplements, good sources of vitamin E are plant-based oils (wheat germ, soybean, and sunflower), nuts (walnuts, peanuts, and almonds), and fruits and vegetables (mango, avocado, asparagus, and red bell pepper) (Table 2).

### 6.2. Curcumin

Curcumin reduces both adiposity and adipose tissue inflammation in mice [41]. Curcumin and white pepper lower high-fat-induced pro-inflammatory cytokines in subcutaneous adipose tissue [42]. The authors state that the effect is independent of adiposity, immune cell recruitment, and gut microbiota changes. A study performed on rats shows that curcumin protects against weight regain and limits adipose tissue growth [43]. Moreover, a study using a rat model for obesity-inducing multi-organ dysfunctionalities supports curcumin’s anti-obesity and anti-inflammatory effects [44]. The weight of epididymal adipose tissue in rats is reduced by curcuminoid intake in a dose-dependent manner [45]. In humans, overweight subjects taking curcumin showed a weight loss and reduction in body fat [46]. Advanced pancreatic cancer patients treated with curcumin had a greater loss of subcutaneous adipose tissue and muscle compared to untreated patients [47]. Curcumin inhibits the NF-κB pathway in adipocytes, reducing cytokine expression and chronic inflammation [48]. Curcumin blocks CCL2-induced adhesion, invasion, and motility of prostate cancer cells [49]. CCL2 expression in plasma and intestinal tissue in a mouse model of colon cancer is decreased by curcumin [50]. A curcumin-supplemented diet lowers the serum levels of CCL5 in rats [52]. Both CCL5 and CCL2 are down-regulated in mouse bone marrow stromal cells by curcumin [51]. CCL7 serum level and lung inflammation are lowered by curcumin [53].

Curcumin inhibits the up-regulation of CXCL1 and CXCL2 induced by 5-fluorouracil in the colon [54]. In breast and prostate cancer, both chemokines are down-regulated by curcumin via NF-κB signaling [55,56]. Another modulator of curcumin’s impact on CXCL1 and 2 levels in breast cancer is miR181b [93]. Curcumin is associated with reduced CXCL10 expression in hepatic tissue [57] and the brain [58]. Curcumin inhibits CXCL12-induced invasion of human esophageal carcinoma cells via the Rac1-PI3K signaling pathway [59]. New curcumin delivery systems act efficiently to suppress the CXCL12/CXCR4 axis and improve the gemcitabine effect on pancreatic cancer [94]. Curcumin weakens endometrial adenocarcinoma cell migration with the down-regulation of CXCL12 via Slit-2 mediation [60].

### 6.3. Flavonoids

Among flavonoids, epicatechin, which is largely present in fruits, acts as an anti-inflammatory on fat tissue by lowering NF-κB pro-inflammatory signals and CCL2 [61] and CCL19 [62] tissue chemokines (Table 2). Epigallocatechin-3-gallate (EGCG), highly present in green tea, reduces obesity and white adipose tissue gain in mice [63,64]. In humans, EGCG in the presence of resveratrol decreases adipogenesis, oxidative stress, and inflammation-related gene expression [65]. Besides its anti-inflammatory effect, EGCG is able to prevent the development of a CAA-like phenotypes in ASCs by inhibiting CCL2, CCL5, immunomodulators (HIF-1α, VEGFα), and NF-κB activation. The same study shows that EGCG hinders the chemotactic response of adipose-derived mesenchymal stem/stromal cells to the triple-negative breast cancer secretome [66].

In a model of obese ovariectomized mice, the flavanone naringenin managed to reduce adiposity and adipose tissue inflammation, and delayed the growth of mammary tumors [67]. The isoflavone genistein has well-recognized anti-obesity and anti-cancer properties. Accordingly, genistein down-regulates CXCL12, lowering the migratory and invasive potential of breast and ovarian cancer cells [72]. In an experimental design using human umbilical vascular endothelial cells stimulated with TNF-alpha, genistein was more efficient than daidzein in decreasing the CCL-2 in a dose-dependent manner [69]. In mice, dietary genistein inhibited TNF-alpha-induced CCL-2 and CXCL8 production [70]. Genistein also blocks the proliferation of melanoma cells by reducing CXCL8 levels [71]. On human synovial fibroblasts, genistein has adipogenic and anti-inflammatory effects. So it is able to reduce both endogenous and TNF-alpha-induced CXCL8 [68].

Quercetin alone or in the presence of green tea extract down-regulates the inflammatory response in adipose tissue of high-fat-diet mice [73]. In humans, a mixed flavonoid–fish oil supplement has an anti-inflammatory effect in obese and overweight women [74]. Tart cherry is presented as an important source of flavonoids such as anthocyanins. Obese rats fed with a diet rich in tart cherry showed a decrease in inflammatory markers in visceral (retroperitoneal and perigonadal) fat, which was not accompanied by changes in visceral fat accumulation [75]. A 2021 study emphasized that tart cherry supplements might have an anti-adipogenic effect by acting directly on the adipose tissue and down-regulating the high-fat-diet-induced mRNA expression of cannabinoid CB1 receptor, peroxisomeproliferator-activated receptor gamma (PPARγ), and sterol regulatory element-binding protein 1c (SREBP-1c) adipogenesis-related genes and transient receptor potential vanilloid subtype 1 (TRPV1) and 2 (TRPV2) channels [76]. Baicalin, a flavonoid extracted from *Scutellaria baicalensis Georgi*, decreases the cell migration induced by chemokines. In order to exert its anti-inflammatory action and limit chemokine activity, baicalin binds to different chemokines (CXCL12, CXCL8, CCL4, and CCL8) [77]. Cirsimarin, a flavonoid obtained from different species such as Cirsium japonicum var. *ussuriense, Cirsium rhothophilum, Cirsium rhinoceros,* and *Microtea debilis*, has anti-lipogenic activity and is able to decrease the intra-abdominal fat in mice by lowering the adipose cell diameter [78]. In the same line, apigenin, abundant in fruits and vegetables, is able to reduce the body weight and visceral fat in obese mice [79].

### 6.4. Organosulfur Components

Another group of food constituents with anti-inflammatory and anti-obesity properties is organosulfur components (Table 2). Vegetables such as garlic, onion, cabbage, cauliflower, and broccoli are excellent sources of organosulfur components. Garlic and onion oils have an anti-obesity effect on rats fed a high-fat diet [29]. Garlic constituents, diallyl disulfide and diallyl trisulfide, have emerged as new therapeutic agents to subdue drug resistance in breast cancer [30]. Diallyl disulfide can suppress the accumulation/activation of macrophages in adipose tissue and inhibit the release of CCL2 from adipocytes lowering the inflammatory status induced by obesity [31]. Diallyl trisulfide prevents adipogenesis in animal models of diet-induced obesity and cultured adipocytes [32,33,34]. Experiments performed on triple-negative breast cancer cells showed that diallyl trisulfide can target CCL2 and other molecules, inducing cell death and inhibiting cell migration [35]. In a pro-inflammatory experimental model, diallyl trisulfide inhibited the activation of CXCL12, showing its anti-chemo-attractive potential [36]. Sulforaphane, another promising compound in lowering obesity and its negative effects [37], is an isothiocyanate present in broccoli, cabbage, cauliflower, and kale [38]. Sulforaphane is able to reduce adipocyte lipid accumulation and lower cytokine production [80].

### 6.5. Fatty Acids

**Fatty acids** are key players in adipose tissue development and function. N-3 FAs have a very well-established beneficial effect on human wellbeing (Table 2). A study using keratinocytes and T cells shows that ALA is able to decrease levels of CCL2, CXCL1, CXCL10, and CXCL8 [81]. In an obesity model comprising rodents fed with ALA-rich flaxseed oil for 8 weeks, the CCL2 level was reduced, as well as adipocyte size and T-cell infiltration in adipose tissue [82]. In another study using a hybrid cell line, EPA and DHA lowered the production of CCL-2 and CXCL-8 [83]. Besides preventing high-fat-diet-induced obesity, EPA in mice is also able to reduce adipose tissue inflammation, adipogenesis, and adipocyte size [84]. In a mouse experimental model of prostate cancer, n-3 FAs lowered the infiltration of macrophages and CCL-2 expression [85]. Experiments using adipocytes generated from human subcutaneous adipose biopsies supported the n-3 FAs lowering CCL-2 levels [86]. CCL-2 and other pro-inflammatory molecules such as IL-6, NFkB, and Ptgs2 have a decreased level in mammary glands of mice fed with n-3 FAs. In these mice, mammary glands and abdominal fat have smaller adipocytes [87]. A co-culture experiment using adipocytes and murine splenic CD8+ T cells has shown that the levels of secreted CCL-3 and CCL-4 are reduced in the presence of n-3 FAs [88]. Conversely, a study using 60% calories from fat with low n-3 and high n-6 (n-6–n-3 ratio of 9:1) showed that this high-fat diet is responsible not only for increased body weight, fat mass, and pancreas weight but also for increased mesenteric adipose tissue and pancreas metaplasia [89].

N-3 fats exert their beneficial role acting on multiple levels, generating a network of interactions. In 2012, Philip Calder published a comprehensive review of n-3 FA mechanisms of action [90]. N-3 FAs can reduce obesity by keeping the systemic and tissue-specific lipid homeostasis under control via transcription factors (NFkB, SERBP, and PPAR) [90,91] and the tricarboxylic acid cycle [92]. Moreover, a well-balanced diet can support the psychophysical wellbeing of cancer patients, especially those being treated with chemotherapy. In this regard, n-3 FAs, along with antioxidants and fiber, may have a beneficial impact on cognitive function and reduce the prevalence of depression and anxiety in colorectal cancer patients [230].

## 7. Conclusions

Obesity is a preventable disease. The treatment approaches should take into consideration that a healthy diet not only promotes weight management but also mitigates the inflammatory processes associated with cancer progression. Dietary changes can significantly improve the overall quality of life for obese cancer patients, reducing treatment-related adverse effects and fostering both physical and mental health. Polyphenols, organosulfur components, and fatty acids are a few categories of active food elements that effectively support and improve human health. Unfortunately, in these times, we are more prone to study real foods than to eat them. So, how can we lower obesity supporting inflammation and metastasis? Besides allopathic, osteopathic, and homeopathic medicine, the new drug combinations and delivery systems, basically what is left to tackle is food intake. There is no magic pill to lower obesity and affect the systemic inflammation associated with it. To maintain a healthy state is a lifelong multi-step process and is beyond the individual (e.g., culinary traditions, life style, race, and ethnicity). Once the disease is present, to undo the damage is an intricate effort. To have a real impact on the prevalence of global obesity and to reduce the obesogenic environment, prevention should address not only the individual (e.g., nutrition and personalized diets, exercising and weight loss medication) but also, and especially, food systems. Food systems are essential to balancing the obesogenic environment with healthy choices. Policy changes should be implemented to lower the sugar content of the foods and to regulate taste enhancers, food colorants, and high-calorie and low-nutrient ultra-processed foods.

## 8. Future Perspectives

Future applications should address the efficiency of different dietary constituents in treating cancer. This might involve the utilization of vitamin E as a complementary therapy alongside conventional treatments, with the objective of enhancing therapeutic efficacy and minimizing the side effects associated with chemotherapy. Moreover, to maximize curcumin efficiency in cancer treatment, new perspectives are required to improve its bioavailability through innovative formulations or delivery systems. Integration of dietary compounds into established cancer treatment protocols could result in more compressive and holistic approaches, such as the development of flavonoid-rich supplements or foods, thus targeting specific cancers for a more personalized treatment approach [231]. It is essential to note that long-term and large-scale clinical trials are necessary to ascertain the efficiency and safety of these compounds in cancer therapy [232,233,234].

## Figures and Tables

**Table 1:** Chemokines released by fat depots.

Chemokine	Fat Depot	High Level Chemokines (Obese vs. Lean)	Where It Can Act	Released by
CCL2 (MCP-1)	Perivascular [11,12]		Invasion/migration Intravasation Circulation Extravasation Colonization	Fibroblasts Macrophages Preadipocytes [13] Adipocytes [13]
	Epicardial [11]			
	Perirenal [11]			
	Subcutaneous [9,11,14,15]	[14,15]		
	Omental [11]			
	Intermuscular [16]			
CCL3 (MIP-1α)	Subcutaneous [9,15]	[15]	Invasion/migration Intravasation Circulation Extravasation Colonization	
	Visceral [9]			
CCL4 (MIP-1β)	Subcutaneous [15]	[15]	Invasion/migration Circulation Extravasation Colonization	
	Pericardial [17]			
CCL5 (Rantes)	Subcutaneous [9,14,15,18]	[14,15]	Invasion/migration	
	Visceral [9,18]			
	Epicardial [18]			
CCL7 (MCP-3)	Subcutaneous [9,14]	[14]	Invasion/migration [19] Intravasation Circulation Colonization	Preadipocytes [13]
	Visceral [9]			
CCL8 (MCP-2)	Subcutaneous [9]		Invasion/migration Intravasation Extravasation Colonization	Preadipocytes [13]
	Visceral [9]			
CCL11 (eotaxin-1)	Subcutaneous [9]		Invasion/migration	Preadipocytes [13]
	Visceral [9]			
CCL18 (MIP-4)	Subcutaneous [15]	[15]	Invasion/migration Intravasation	Adipocytes [13]
CCL19 (MIP-3 β)	Subcutaneous [14,20]	[14,20]	Invasion/migration	Adipocytes [13]
CCL20 (MIP-3 α; LARC)	Subcutaneous [15]	[15]	Invasion/migration Intravasation Colonization	
CXCL1 (Gro α)	Subcutaneous [14]	[14]	Invasion/migration Intravasation Colonization	Preadipocytes [13] Adipocytes [13]
CXCL2 (Gro β)	Omentum [21]		Proliferation/migration/angiogenesis	Adipocytes [21]
CXCL5 (ENA78)	Subcutaneous [14]	[14]	Invasion/migration Colonization	
CXCL8 (IL-8)	Subcutaneous [14]	[14]	Invasion/migration Colonization	
	Perivascular [12]			
CXCL10 (IP-10)	Subcutaneous [14]	[14]	Invasion/migration Colonization	Preadipocytes [13] Adipose stem cells [22]
	Omentum [22]			
CXCL12 (SDF-1)			Invasion/migration Colonization	Adipocytes [23] Adipose stromal cells [24]
CCR1 (CD191)	Subcutaneous [9]		Invasion/migration	
	Visceral [9]			
CCR2 (CD192)	Subcutaneous [9]		Invasion/migration	
	Visceral [9]			
CCR3 (CD193)	Subcutaneous [9]		Invasion/migration	
	Visceral [9]			
CCR5 (CD195)	Subcutaneous [9]		Invasion/migration	Adipose stem cells [22]
	Visceral [9]			
	Omentum [22]			

**Table 2:** Nutrients **lowering** (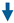) adiposity and chemokine release.

Nutrients	Source	Effect on Adipose Tissue	
**Viamin E**	Plant-based oils (wheat-germ, soybean, sun flower); Nuts (walnuts, peanuts, almonds); Fruits and vegetables	CCL2 [39] NF-κB [40]	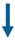
**Curcumin**	Rhizome Curcuma longa	Adiposity and adipose tissue inflammation [41,42,43,44,45,46,47], NF-κB [48], CCL2 [49,50,51], CCL5 [51,52], CCL7 [53], CXCL1 and CXCL2 [54,55,56], CXCL10 [57,58], CXCL12 [59,60]	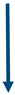
**Flavonoids**			
Epicatechin	Fruits	Adipose tissue inflammation [61,62], NF-κB [61], CCL2 [61], CCL19 [62]	
Epigallocatechin-3- gallate	Green tea	Adipogenesis [63,64,65], NF-κB, CCL2 and CCL5 [66]	
Naringenin	Citrus fruits, tart cherries, tomatoes	Adiposity and adipose tissue inflammation [67]	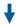
Genistein	Soy beans, soy-derived foods	Adipose tissue inflammation [68], CCL2 [69,70], CXCL8 [68,70,71], CXCL12 [72]	
Quercetin	Fruits and vegetables	Adipose tissue inflammation [73]	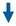
Flavonoid fish-oil supplement		Adipose tissue inflammation [74]	
Anthocyanins	Tart cherry	Adipose tissue inflammation [75], adipogenesis [76]	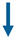
Baicalin	*Scutellaria baicalensis Georgi*	Adipose tissue inflammation, chemokine activity [77]	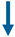
Cirsimarin	*Cirsium japonicum* var. *ussuriense*, *Cirsium rhothophilum*, *Cirsium rhinoceros*, *Microtea debilis*	Lipogenesis, adipose cell diameter [78]	
Apigenin	Fruits and vegetables	Adipogenesis [79]	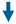
**Organosulfur** **components**			
Diallyl disulfide	Garlic	Obesity [29], adipose tissue inflammation [31], CCL2 [31]	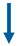
Diallyl trisulfide	Garlic	Adipogenesis [32,33,34], CCL2 [35], CXCL12 [36]	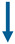
Sulforaphane	Broccoli, cabbage, cauliflower, kale	Obesity [37], adipocyte lipid accumulation [80], cytokine production [80]	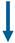
**Fatty acids**			
n-3 FAs: ALA, EPA, DHA	Fish, nuts, seeds, oils (canola, soybean)	Adipose cell size; NF-κB; CCL2, CCL3, CCL4, CXCL1, CXCL10, CXCL8 [81,82,83,84,85,86,87,88,89,90,91,92]	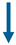

## Abbreviations

The following abbreviations are used in this manuscript:

CDC	Centers for Disease Control and Prevention
JNK	c-jun N-terminal kinase
CCL	chemokine (C-C motif) ligand
CXCL	C-X-C motif chemokine ligand
CCR	C-C chemokine receptor type
n-3 Fas	omega-3 polyunsaturated fatty acids
ALA	α-linolenic acid
EPA	eicosapentaenoic acid
DHA	docosahexaenoic acid
CAA	cancer-associated adipocytes
EGCG	epigallocatechin-3-gallate
MIP	macrophage Inflammatory Proteins
MCP1	monocyte chemoattractant protein 1
RANTES	regulated on activation, normal T-cell expressed and secreted
MDSCs	monocytic myeloid-derived suppressor cells
Groα, Gro1	growth-regulated protein alpha
MGSA-α	melanoma growth-stimulating activity-alpha
SDF-1	stromal cell-derived factor 1
NF-κB	nuclear factor kappa-light-chain-enhancer of activated B cells
SRY	Sex-determining region Y
HIF-1α	hypoxia-inducible factor-1a
VEGF-A	vascular endothelial growth factor A
Her2	human epidermal growth factor receptor 2
ASC	adipose stromal/stem cells
ALDOC	aldolase, fructose-bisphosphate C
*ENO2*	enolase 2
HK1, 2	hexokinase
PFKP	phosphofructokinase, platelet
CAA	cancer-associated adipocytes
WAT	white adipose tissue
VEGF	vascular endothelial growth factor
ERK	extracellular signal-regulated kinase
ELK1	ETS domain transcription factor 1
EGR1	early Growth Response 1
STAT3	signal transducer and activator of transcription 3
JAK2	Janus kinase 2
TNF	tumor necrosis factor
CEBPB	CCAAT/enhancer-binding protein bet
EGF	epidermal growth factor

## References

[1] https://www.cdc.gov/cancer/risk-factors/obesity.html.

[2] Lega I.C., Lipscombe L.L. (2020). Review: Diabetes, Obesity, and Cancer—Pathophysiology and Clinical Implications. Endocr. Rev..

[3] Deng T., Lyon C.J., Bergin S., Caligiuri M.A., Hsueh W.A. (2016). Obesity, Inflammation, and Cancer. Annu. Rev. Pathol. Mech. Dis..

[4] Zhang Y., Liu J., Yao J., Ji G., Qian L., Wang J., Zhang G., Tian J., Nie Y., Zhang Y.E. (2014). Obesity: Pathophysiology and Intervention. Nutrients.

[5] Stienstra R., Tack C.J., Kanneganti T.D., Joosten L.A.B., Netea M.G. (2012). The Inflammasome Puts Obesity in the Danger Zone. Cell Metab..

[6] Gregor M.F., Hotamisligil G.S. (2011). Inflammatory Mechanisms in Obesity. Annu. Rev. Immunol..

[7] Ramos-Nino M.E. (2013). The Role of Chronic Inflammation in Obesity-Associated Cancers. ISRN Oncol..

[8] Stone T.W., McPherson M., Gail Darlington L. (2018). Obesity and Cancer: Existing and New Hypotheses for a Causal Connection. EBioMedicine.

[9] Huber J., Kiefer F.W., Zeyda M., Ludvik B., Silberhumer G.R., Prager G., Zlabinger G.J., Stulnig T.M. (2008). CC Chemokine and CC Chemokine Receptor Profiles in Visceral and Subcutaneous Adipose Tissue Are Altered in Human Obesity. J. Clin. Endocrinol. Metab..

[10] Lim S.Y., Yuzhalin A.E., Gordon-Weeks A.N., Muschel R.J. (2016). Targeting the CCL2-CCR2 signaling axis in cancer metastasis. Oncotarget.

[11] Chatterjee T.K., Stoll L.L., Denning G.M., Harrelson A., Blomkalns A.L., Idelman G., Rothenberg F.G., Neltner B., Romig-Martin S.A., Dickson E.W. (2009). Proinflammatory Phenotype of Perivascular Adipocytes. Circ. Res..

[12] Henrichot E., Juge-Aubry C.E., Pernin A., Pache J.C., Velebit V., Dayer J.M., Meda P., Chizzolini C., Meier C.A. (2005). Production of Chemokines by Perivascular Adipose Tissue. Arterioscler. Thromb. Vasc. Biol..

[13] Ignacio R.M.C., Gibbs C.R., Lee E.S., Son D.S. (2016). Differential Chemokine Signature between Human Preadipocytes and Adipocytes. Immune Netw..

[14] Tourniaire F., Romier-Crouzet B., Lee J.H., Marcotorchino J., Gouranton E., Salles J., Malezet C., Astier J., Darmon P., Blouin E. (2013). Chemokine Expression in Inflamed Adipose Tissue Is Mainly Mediated by NF-κB. PLoS ONE.

[15] Kopasov A.E., Blokhin S.N., Volkova E.N., Morozov S.G. (2019). Chemokine Expression in Neutrophils and Subcutaneous Adipose Tissue Cells Obtained during Abdominoplasty from Patients with Obesity and Normal Body Weight. Bull. Exp. Biol. Med..

[16] Haam J.H., Kim Y.S., Koo H.S., Haam J., Seo N.K., Kim H.Y., Park K.C., Park K.S., Kim M.J. (2016). Intermuscular adipose tissue is associated with monocyte chemoattractant protein-1, independent of visceral adipose tissue. Clin. Biochem..

[17] Lau F.H., Deo R.C., Mowrer G., Caplin J., Ahfeldt T., Kaplan A., Ptaszek L., Walker J.D., Rosengard B.R., Cowan C.A. (2011). Pattern Specification and Immune Response Transcriptional Signatures of Pericardial and Subcutaneous Adipose Tissue. PLoS ONE.

[18] Madani R., Karastergiou K., Ogston N.C., Miheisi N., Bhome R., Haloob N., Tan G.D., Karpe F., Malone-Lee J., Hashemi M. (2009). RANTES release by human adipose tissue in vivo and evidence for depot-specific differences. Am. J. Physiol. Metab..

[19] Liu Y., Cai Y., Liu L., Wu Y., Xiong X. (2018). Crucial biological functions of CCL7 in cancer. Peer J..

[20] Kochumon S., Al-Rashed F., Abu-Farha M., Devarajan S., Tuomilehto J., Ahmad R. (2019). Adipose tissue expression of CCL19 chemokine is positively associated with insulin resistance. Diabetes Metab. Res. Rev..

[21] Natsume M., Shimura T., Iwasaki H., Okuda Y., Hayashi K., Takahashi S., Kataoka H. (2020). Omental adipocytes promote peritoneal metastasis of gastric cancer through the CXCL2–VEGFA axis. Br. J. Cancer.

[22] Ghaderi A., Rezaeifard S., Razmkhah M., Robati M., Momtahan M. (2014). Adipose derived stem cells isolated from omentum: A novel source of chemokines for ovarian cancer growth. J. Cancer Res. Ther..

[23] Kim D., Kim J., Yoon J.H., Ghim J., Yea K., Song P., Park S., Lee A., Hong C.P., Jang M.S. (2014). CXCL12 secreted from adipose tissue recruits macrophages and induces insulin resistance in mice. Diabetologia.

[24] Saha A., Hamilton-Reeves J., DiGiovanni J. (2022). White adipose tissue-derived factors and prostate cancer progression: Mechanisms and targets for interventions. Cancer Metastasis Rev..

[25] Wu Y., Li Y.Y., Matsushima K., Baba T., Mukaida N. (2008). CCL3-CCR5 Axis Regulates Intratumoral Accumulation of Leukocytes and Fibroblasts and Promotes Angiogenesis in Murine Lung Metastasis Process. J. Immunol..

[26] Silva T.A., Ribeiro F.L.L., de Oliveira-Neto H.H., Watanabe S., De Cassia Gonçalves Alencar R., Fukada S.Y., Queiroz Cunha F., Rodrigues Leles C., Mendonça E.F., Carvalho Batista A. (2007). Dual role of CCL3/CCR1 in oral squamous cell carcinoma: Implications in tumor metastasis and local host defense. Oncol. Rep..

[27] Mincione G., Pagnini P., Lenzi G. (1964). Histological study of the hypophysis, thyroid gland, adrenal cortex and ovary in “adenocirrhosis” and cancer cirrhosis due to CCL-4 and hepatoma due to p-dimethylaminoazobence in the rat. Arch. De. Vecchi Anat. Patol..

[28] Mincione G., Pagnini P. (1964). Study of ceroid and siderinic pigments in experimental liver cirrhosis due to CCL4, in adenocirrhosis and in cancrocirrhosis due to CCL4 and p-dimethylaminoazobenzene. Arch. De. Vecchi Anat. Patol..

[29] Yang C., Li L., Yang L., Lǚ H., Wang S., Sun G. (2018). Anti-obesity and Hypolipidemic effects of garlic oil and onion oil in rats fed a high-fat diet. Nutr. Metab..

[30] Malla R., Marni R., Chakraborty A., Kamal M.A. (2022). Diallyl disulfide and diallyl trisulfide in garlic as novel therapeutic agents to overcome drug resistance in breast cancer. J. Pharm. Anal..

[31] Woo H.M., Kang J.H., Kawada T., Yoo H., Sung M.K., Yu R. (2007). Active spice-derived components can inhibit inflammatory responses of adipose tissue in obesity by suppressing inflammatory actions of macrophages and release of monocyte chemoattractant protein-1 from adipocytes. Life Sci..

[32] Miura A., Ikeda A., Abe M., Seo K., Watanabe T., Ozaki-Masuzawa Y., Hosono T., Seki T. (2021). Diallyl Trisulfide Prevents Obesity and Decreases miRNA-335 Expression in Adipose Tissue in a Diet-Induced Obesity Rat Model. Mol. Nutr. Food Res..

[33] Hu Y., Xu J., Gao R., Xu Y., Huangfu B., Asakiya C., Huang X., Zhang F., Huang K., He X. (2022). Diallyl Trisulfide Prevents Adipogenesis and Lipogenesis by Regulating the Transcriptional Activation Function of KLF15 on PPARγ to Ameliorate Obesity. Mol. Nutr. Food Res..

[34] Lii C.K., Huang C.Y., Chen H.W., Chow M.Y., Lin Y.R., Huang C.S., Tsai C.W. (2012). Diallyl trisulfide suppresses the adipogenesis of 3T3-L1 preadipocytes through ERK activation. Food Chem. Toxicol..

[35] Kanga K.J.W., Mendonca P., Soliman K.F.A., Ferguson D.T., Darling-Reed S.F. (2021). Effect of Diallyl Trisulfide on TNF-α-induced CCL2/MCP-1 Release in Genetically Different Triple-negative Breast Cancer Cells. Anticancer Res..

[36] Lee H.H., Jeong J.W., Hong S.H., Park C., Kim B.W., Choi Y.H. (2018). Diallyl Trisulfide Suppresses the Production of Lipopolysaccharide-induced Inflammatory Mediators in BV2 Microglia by Decreasing the NF-κB Pathway Activity Associated with Toll-like Receptor 4 and CXCL12/CXCR4 Pathway Blockade. J. Cancer Prev..

[37] Martins T., Colaço B., Venâncio C., Pires M.J., Oliveira P.A., Rosa E., Antunes L.M. (2018). Potential effects of sulforaphane to fight obesity. J. Sci. Food Agric..

[38] Vanduchova A., Anzenbacher P., Anzenbacherova E. (2019). Isothiocyanate from Broccoli, Sulforaphane, and Its Properties. J. Med. Food.

[39] Lin Y., Huang R., Santanam N., Liu Y.G., Parthasarathy S., Huang R.P. (2002). Profiling of human cytokines in healthy individuals with vitamin E supplementation by antibody array. Cancer Lett..

[40] Matsunaga T., Shoji A., Gu N., Joo E., Li S., Adachi T., Yamazaki H., Yasuda K., Kondoh T., Tsuda K. (2012). γ-tocotrienol attenuates TNF-α-induced changes in secretion and gene expression of MCP-1, IL-6 and adiponectin in 3T3-L1 adipocytes. Mol. Med. Rep..

[41] Islam T., Koboziev I., Albracht-Schulte K., Mistretta B., Scoggin S., Yosofvand M., Moussa H., Zabet-Moghaddam M., Ramalingam L., Gunaratne P.H. (2021). Curcumin Reduces Adipose Tissue Inflammation and Alters Gut Microbiota in Diet-Induced Obese Male Mice. Mol. Nutr. Food Res..

[42] Neyrinck A.M., Alligier M., Memvanga P.B., Névraumont E., Larondelle Y., Préat V., Cani P.D., Delzenne N.M. (2013). Curcuma longa Extract Associated with White Pepper Lessens High Fat Diet-Induced Inflammation in Subcutaneous Adipose Tissue. PLoS ONE.

[43] Teich T., Pivovarov J.A., Porras D.P., Dunford E.C., Riddell M.C. (2017). Curcumin limits weight gain, adipose tissue growth, and glucose intolerance following the cessation of exercise and caloric restriction in rats. J. Appl. Physiol..

[44] Hassan M.H., Awadalla E.A., Abd El-Kader A.E.K.M., Seifeldin E.A., Mahmoud M.A., Muddathir A.R.M., Abdelsadik A. (2022). Antitoxic Effects of Curcumin against Obesity-Induced Multi-Organs’ Biochemical and Histopathological Abnormalities in an Animal Model. Evidence-Based Complement. Altern. Med..

[45] Asai A., Miyazawa T. (2001). Dietary Curcuminoids Prevent High-Fat Diet–Induced Lipid Accumulation in Rat Liver and Epididymal Adipose Tissue. J. Nutr..

[46] Di Pierro F., Bressan A., Ranaldi D., Rapacioli G., Giacomelli L., Bertuccioli A. (2015). Potential role of bioavailable curcumin in weight loss and omental adipose tissue decrease: Preliminary data of a randomized, controlled trial in overweight people with metabolic syndrome. Preliminary study. Eur. Rev. Med. Pharmacol. Sci..

[47] Parsons H.A., Baracos V.E., Hong D.S., Abbruzzese J., Bruera E., Kurzrock R. (2016). The effects of curcumin (diferuloylmethane) on body composition of patients with advanced pancreatic cancer. Oncotarget.

[48] Gonzales A.M., Orlando R.A. (2008). Curcumin and resveratrol inhibit nuclear factor-kappaB-mediated cytokine expression in adipocytes. Nutr. Metab..

[49] Herman J.G., Stadelman H.L., Roselli C.E. (2009). Curcumin blocks CCL2-induced adhesion, motility and invasion, in part, through down-regulation of CCL2 expression and proteolytic activity. Int. J. Oncol..

[50] Murphy E.A., Davis J.M., McClellan J.L., Gordon B.T., Carmichael M.D. (2011). Curcumin’s Effect on Intestinal Inflammation and Tumorigenesis in the Apc Min/+ Mouse. J. Interf. Cytokine Res..

[51] Xu Y.X., Pindolia K.R., Janakiraman N., Noth C.J., Chapman R.A., Gautam S.C. (1997). Curcumin, a compound with anti-inflammatory and anti-oxidant properties, down-regulates chemokine expression in bone marrow stromal cells. Exp. Hematol..

[52] Pickich M.B., Hargrove M.W., Phillips C.N., Healy J.C., Moore A.N., Roberts M.D., Martin J.S. (2019). Effect of curcumin supplementation on serum expression of select cytokines and chemokines in a female rat model of nonalcoholic steatohepatitis. BMC Res. Notes.

[53] Wang Y., Wang Y., Cai N., Xu T., He F. (2021). Anti-inflammatory effects of curcumin in acute lung injury: In vivo and in vitro experimental model studies. Int. Immunopharmacol..

[54] Sakai H., Kai Y., Oguchi A., Kimura M., Tabata S., Yaegashi M., Saito T., Sato K., Sato F., Yumoto T. (2016). Curcumin Inhibits 5-Fluorouracil-induced Up-regulation of CXCL1 and CXCL2 of the Colon Associated with Attenuation of Diarrhoea Development. Basic. Clin. Pharmacol. Toxicol..

[55] Bachmeier B.E., Mohrenz I.V., Mirisola V., Schleicher E., Romeo F., Höhneke C., Jochum M., Nerlich A.G., Pfeffer U. (2008). Curcumin downregulates the inflammatory cytokines CXCL1 and -2 in breast cancer cells via NFκB. Carcinogenesis.

[56] Killian P.H., Kronski E., Michalik K.M., Barbieri O., Astigiano S., Sommerhoff C.P., Pfeffer U., Nerlich A.G., Bachmeier B.E. (2012). Curcumin inhibits prostate cancer metastasis in vivo by targeting the inflammatory cytokines CXCL1 and -2. Carcinogenesis.

[57] Tu C., Han B., Liu H., Zhang S. (2011). Curcumin protects mice against concanavalin A-induced hepatitis by inhibiting intrahepatic intercellular adhesion molecule-1 (ICAM-1) and CXCL10 expression. Mol. Cell Biochem..

[58] Dende C., Meena J., Nagarajan P., Nagaraj V.A., Panda A.K., Padmanaban G. (2017). Nanocurcumin is superior to native curcumin in preventing degenerative changes in Experimental Cerebral Malaria. Sci. Rep..

[59] Lin M.L., Lu Y.C., Chen H.Y., Lee C.C., Chung J.G., Chen S.S. (2014). Suppressing the formation of lipid raft-associated Rac1/PI3K/Akt signaling complexes by curcumin inhibits SDF-1α-induced invasion of human esophageal carcinoma cells. Mol. Carcinog..

[60] Sirohi V.K., Popli P., Sankhwar P., Kaushal J.B., Gupta K., Manohar M., Dwivedi A. (2017). Curcumin exhibits anti-tumor effect and attenuates cellular migration via Slit-2 mediated down-regulation of SDF-1 and CXCR4 in endometrial adenocarcinoma cells. J. Nutr. Biochem..

[61] Bettaieb A., Cremonini E., Kang H., Kang J., Haj F.G., Oteiza P.I. (2016). Anti-inflammatory actions of (−)-epicatechin in the adipose tissue of obese mice. Int. J. Biochem. Cell Biol..

[62] Sano T., Nagayasu S., Suzuki S., Iwashita M., Yamashita A., Shinjo T., Sanui T., Kushiyama A., Kanematsu T., Asano T. (2017). Epicatechin downregulates adipose tissue CCL19 expression and thereby ameliorates diet-induced obesity and insulin resistance. Nutr. Metab. Cardiovasc. Dis..

[63] Bose M., Lambert J.D., Ju J., Reuhl K.R., Shapses S.A., Yang C.S. (2008). The Major Green Tea Polyphenol, (-)-Epigallocatechin-3-Gallate, Inhibits Obesity, Metabolic Syndrome, and Fatty Liver Disease in High-Fat–Fed Mice. J. Nutr..

[64] Li F., Gao C., Yan P., Zhang M., Wang Y., Hu Y., Wu X., Wang X., Sheng J. (2018). EGCG Reduces Obesity and White Adipose Tissue Gain Partly Through AMPK Activation in Mice. Front. Pharmacol..

[65] Most J., Warnke I., Boekschoten M.V., Jocken J.W.E., de Groot P., Friedel A., Bendik I., Goossens G.H., Blaak E.E. (2018). The effects of polyphenol supplementation on adipose tissue morphology and gene expression in overweight and obese humans. Adipocyte.

[66] Gonzalez Suarez N., Fernandez-Marrero Y., Torabidastgerdooei S., Annabi B. (2022). EGCG Prevents the Onset of an Inflammatory and Cancer-Associated Adipocyte-like Phenotype in Adipose-Derived Mesenchymal Stem/Stromal Cells in Response to the Triple-Negative Breast Cancer Secretome. Nutrients.

[67] Ke J.Y., Banh T., Hsiao Y.H., Cole R.M., Straka S.R., Yee L.D., Belury M.A. (2017). Citrus flavonoid naringenin reduces mammary tumor cell viability, adipose mass, and adipose inflammation in obese ovariectomized mice. Mol. Nutr. Food Res..

[68] Relic B., Zeddou M., Desoroux A., Beguin Y., de Seny D., Malaise M.G. (2009). Genistein induces adipogenesis but inhibits leptin induction in human synovial fibroblasts. Lab. Investig..

[69] Cho H.Y., Park C.M., Kim M.J., Chinzorig R., Cho C.W., Song Y.S. (2011). Comparative effect of genistein and daidzein on the expression of MCP-1, eNOS, and cell adhesion molecules in TNF-α-stimulated HUVECs. Nutr. Res. Pract..

[70] Jia Z., Babu P.V.A., Si H., Nallasamy P., Zhu H., Zhen W., Misra H.P., Li Y., Liu D. (2013). Genistein inhibits TNF-α-induced endothelial inflammation through the protein kinase pathway A and improves vascular inflammation in C57BL/6 mice. Int. J. Cardiol..

[71] Venza I., Visalli M., Oteri R., Beninati C., Teti D., Venza M. (2018). Genistein reduces proliferation of EP3-expressing melanoma cells through inhibition of PGE2-induced IL-8 expression. Int. Immunopharmacol..

[72] Hsu E.L., Chen N., Westbrook A., Wang F., Zhang R., Taylor R.T., Hankinson O. (2009). Modulation of CXCR4, CXCL12, and Tumor Cell Invasion Potential In Vitro by Phytochemicals. J. Oncol..

[73] Cialdella-Kam L., Ghosh S., Meaney M., Knab A., Shanely R., Nieman D. (2017). Quercetin and Green Tea Extract Supplementation Downregulates Genes Related to Tissue Inflammatory Responses to a 12-Week High Fat-Diet in Mice. Nutrients.

[74] Cialdella-Kam L., Nieman D.C., Knab A.M., Shanely R.A., Meaney M.P., Jin F., Sha W., Ghosh S. (2016). A Mixed Flavonoid-Fish Oil Supplement Induces Immune-Enhancing and Anti-Inflammatory Transcriptomic Changes in Adult Obese and Overweight Women—A Randomized Controlled Trial. Nutrients.

[75] Moruzzi M., Klöting N., Blüher M., Martinelli I., Tayebati S.K., Gabrielli M.G., Roy P., Micioni Di Bonaventura M.V., Cifani C., Lupidi G. (2021). Tart Cherry Juice and Seeds Affect Pro-Inflammatory Markers in Visceral Adipose Tissue of High-Fat Diet Obese Rats. Molecules.

[76] Cocci P., Moruzzi M., Martinelli I., Maggi F., Micioni Di Bonaventura M.V., Cifani C., Mosconi G., Tayebati S.K., Damiano S., Lupidi G. (2021). Tart cherry (*Prunus cerasus* L.) dietary supplement modulates visceral adipose tissue CB1 mRNA levels along with other adipogenesis-related genes in rat models of diet-induced obesity. Eur. J. Nutr..

[77] Li B.Q., Fu T., Gong W.H., Dunlop N., Kung H., Yan Y., Kang J., Wang J.M. (2000). The flavonoid baicalin exhibits anti-inflammatory activity by binding to chemokines. Immunopharmacology.

[78] Zarrouki B., Pillon N.J., Kalbacher E., Soula H.A., Nia N’Jomen G., Grand L., Chambert S., Geloen A., Soulage C.O. (2010). Cirsimarin, a potent antilipogenic flavonoid, decreases fat deposition in mice intra-abdominal adipose tissue. Int. J. Obes..

[79] Su T., Huang C., Yang C., Jiang T., Su J., Chen M., Fatima S., Gong R., Hu X., Bian Z. (2020). Apigenin inhibits STAT3/CD36 signaling axis and reduces visceral obesity. Pharmacol. Res..

[80] Folkard D. (2014). Suppression of pro-inflammatory signalling pathways by sulforaphane. Ph.D Thesis.

[81] Morin S., Simard M., Rioux G., Julien P., Pouliot R. (2022). Alpha-Linolenic Acid Modulates T Cell Incorporation in a 3D Tissue-Engineered Psoriatic Skin Model. Cells.

[82] Baranowski M., Enns J., Blewett H., Yakandawala U., Zahradka P., Taylor C.G. (2012). Dietary flaxseed oil reduces adipocyte size, adipose monocyte chemoattractant protein-1 levels and T-cell infiltration in obese, insulin-resistant rats. Cytokine.

[83] Baker E.J., Valenzuela C.A., De Souza C.O., Yaqoob P., Miles E.A., Calder P.C. (2020). Comparative anti-inflammatory effects of plant- and marine-derived omega-3 fatty acids explored in an endothelial cell line. Biochim. Biophys. Acta Mol. Cell Biol. Lipids.

[84] LeMieux M.J., Kalupahana N.S., Scoggin S., Moustaid-Moussa N. (2015). Eicosapentaenoic Acid Reduces Adipocyte Hypertrophy and Inflammation in Diet-Induced Obese Mice in an Adiposity-Independent Manner. J. Nutr..

[85] Liang P., Henning S.M., Schokrpur S., Wu L., Doan N., Said J., Grogan T., Elashoff D., Cohen P., Aronson W.J. (2016). Effect of Dietary Omega-3 Fatty Acids on Tumor-Associated Macrophages and Prostate Cancer Progression. Prostate.

[86] Ferguson J.F., Roberts-Lee K., Borcea C., Smith H.M., Midgette Y., Shah R. (2019). Omega-3 polyunsaturated fatty acids attenuate inflammatory activation and alter differentiation in human adipocytes. J. Nutr. Biochem..

[87] Khadge S., Thiele G.M., Sharp J.G., McGuire T.R., Klassen L.W., Black P.N., DiRusso C., Talmadge J. (2018). Long-Chain Omega-3 Polyunsaturated Fatty Acids Modulate Mammary Gland Composition and Inflammation. J. Mammary Gland. Biol. Neoplasia.

[88] Monk J.M., Liddle D.M., De Boer A.A., Brown M.J., Power K.A., Ma D.W., Robinson L. (2015). Fish-Oil–Derived n–3 PUFAs Reduce Inflammatory and Chemotactic Adipokine-Mediated Cross-talk between Co-cultured Murine Splenic CD8+ T Cells and Adipocytes. J. Nutr..

[89] Ead A.S., Wirkus J., Matsukuma K., Mackenzie G.G. (2024). A high-fat diet induces changes in mesenteric adipose tissue accelerating early-stage pancreatic carcinogenesis in mice. J. Nutr. Biochem..

[90] Calder P.C. (2012). Mechanisms of Action of (n-3) Fatty Acids. J. Nutr..

[91] Deckelbaum R.J., Worgall T.S., Seo T. (2006). n−3 Fatty acids and gene expression. Am. J. Clin. Nutr..

[92] Liu R., Chen L., Wang Z., Zheng X., Hou Z., Zhao D., Long J., Liu J. (2021). Omega-3 polyunsaturated fatty acids prevent obesity by improving tricarboxylic acid cycle omeostasis. J. Nutr. Biochem..

[93] Kronski E., Fiori M.E., Barbieri O., Astigiano S., Mirisola V., Killian P.H., Bruno A., Pagani A., Rovera A., Pfeffer U. (2014). miR181b is induced by the chemopreventive polyphenol curcumin and inhibits breast cancer metastasis via down-regulation of the inflammatory cytokines CXCL1 and -2. Mol. Oncol..

[94] Khan S., Setua S., Kumari S., Dan N., Massey A., Hafeez B.B., Yallapu M.M., Stiles Z.E., Alabkaa A., Yue J. (2019). Superparamagnetic iron oxide nanoparticles of curcumin enhance gemcitabine therapeutic response in pancreatic cancer. Biomaterials.

[95] Liput K.P., Lepczyński A., Ogłuszka M., Nawrocka A., Poławska E., Grzesiak A., Ślaska B., Pareek C.S., Czarnik U., Pierzchała M. (2021). Effects of Dietary n–3 and n–6 Polyunsaturated Fatty Acids in Inflammation and Cancerogenesis. Int. J. Mol. Sci..

[96] Poggioli R., Hirani K., Jogani V.G., Ricordi C. (2023). Modulation of inflammation and immunity by omega-3 fatty acids: A possible role for prevention and to halt disease progression in autoimmune, viral, and age-related disorders. Eur. Rev. Med. Pharmacol. Sci..

[97] Akinsete J.A., Ion G., Witte T.R., Hardman W.E. (2012). Consumption of high ω-3 fatty acid diet suppressed prostate tumorigenesis in C3(1) Tag mice. Carcinogenesis.

[98] Ion G., Akinsete J.A., Hardman W.E. (2010). Maternal consumption of canola oil suppressed mammary gland tumorigenesis in C3(1) TAg mice offspring. BMC Cancer.

[99] Ion G., Akinsete J.A., Witte T.R., Bostan M., Hardman W.E. (2021). Maternal fish oil consumption has a negative impact on mammary gland tumorigenesis in C3(1) Tag mice offspring. Eur. J. Nutr..

[100] AL-Jawadi A., Moussa H., Ramalingam L., Dharamawardhane S., Gollahon L., Gunaratne P., Rahman R.L., Moustaid-Moussa N. (2018). Protective properties of n-3 fatty acids and implications in obesity-associated breast cancer. J. Nutr. Biochem..

[101] Tao X., Zhou Q., Rao Z. (2022). Efficacy of ω-3 Polyunsaturated Fatty Acids in Patients with Lung Cancer Undergoing Radiotherapy and Chemotherapy: A Meta-Analysis. Int. J. Clin. Pract..

[102] Aldoori J., Cockbain A.J., Toogood G.J., Hull M.A. (2022). Omega-3 polyunsaturated fatty acids: Moving towards precision use for prevention and treatment of colorectal cancer. Gut.

[103] Lu Y., Li D., Wang L., Zhang H., Jiang F., Zhang R., Xu L., Yang N., Dai S., Xu X. (2023). Comprehensive Investigation on Associations between Dietary Intake and Blood Levels of Fatty Acids and Colorectal Cancer Risk. Nutrients.

[104] Chait A., den Hartigh L.J. (2020). Adipose Tissue Distribution, Inflammation and Its Metabolic Consequences, Including Diabetes and Cardiovascular Disease. Front. Cardiovasc. Med..

[105] Jensen M.D. (2008). Role of Body Fat Distribution and the Metabolic Complications of Obesity. J. Clin. Endocrinol. Metab..

[106] Sacks H., Symonds M.E. (2013). Anatomical Locations of Human Brown Adipose Tissue. Diabetes.

[107] Leitner B.P., Huang S., Brychta R.J., Duckworth C.J., Baskin A.S., McGehee S., Tal I., Dieckmann W., Gupta G., Kolodny G.M. (2017). Mapping of human brown adipose tissue in lean and obese young men. Proc. Natl. Acad. Sci. USA.

[108] Kim J.W., Kim J.H., Lee Y.J. (2024). The Role of Adipokines in Tumor Progression and Its Association with Obesity. Biomedicines.

[109] Groves D.T., Jiang Y. (1995). Chemokines, a Family of Chemotactic Cytokines. Crit. Rev. Oral. Biol. Med..

[110] Nagarsheth N., Wicha M.S., Zou W. (2017). Chemokines in the cancer microenvironment and their relevance in cancer immunotherapy. Nat. Rev. Immunol..

[111] Fu X., Wang Q., Du H., Hao H. (2023). CXCL8 and the peritoneal metastasis of ovarian and gastric cancer. Front. Immunol..

[112] Łukaszewicz-Zając M., Pączek S., Muszyński P., Kozłowski M., Mroczko B. (2019). Comparison between clinical significance of serum CXCL-8 and classical tumor markers in oesophageal cancer (OC) patients. Clin. Exp. Med..

[113] Pawluczuk E., Łukaszewicz-Zając M., Gryko M., Kulczyńska-Przybik A., Mroczko B. (2021). Serum CXCL8 and Its Specific Receptor (CXCR2) in Gastric Cancer. Cancers.

[114] Feng L., Qi Q., Wang P., Chen H., Chen Z., Meng Z., Liu L. (2018). Serum levels of IL-6, IL-8, and IL-10 are indicators of prognosis in pancreatic cancer. J. Int. Med. Res..

[115] Milovanović J., Todorović-Raković N., Radulovic M. (2019). Interleukin-6 and interleukin-8 serum levels in prognosis of hormone-dependent breast cancer. Cytokine.

[116] Bi L.K., Zhou N., Liu C., Lu F.D., Lin T.X., Xuan X.J., Jiang C., Han J.L., Huang H., Zhang C.X. (2014). Kidney cancer cells secrete IL-8 to activate Akt and promote migration of mesenchymal stem cells. Urol. Oncol. Semin. Orig. Investig..

[117] Chan P.C., Lu C.H., Chien H.C., Tian Y.F., Hsieh P.S. (2022). Adipose Tissue-Derived CCL5 Enhances Local Pro-Inflammatory Monocytic MDSCs Accumulation and Inflammation via CCR5 Receptor in High-Fat Diet-Fed Mice. Int. J. Mol. Sci..

[118] You Y., Li Y., Li M., Lei M., Wu M., Qu Y., Yuan Y., Chen T., Jiang H. (2017). Ovarian cancer stem cells promote tumour immune privilege and invasion via CCL5 and regulatory T cells. Clin. Exp. Immunol..

[119] Huang R., Wang S., Wang N., Zheng Y., Zhou J., Yang B., Wang X., Zhang J., Guo L., Wang S. (2020). CCL5 derived from tumor-associated macrophages promotes prostate cancer stem cells and metastasis via activating β-catenin/STAT3 signaling. Cell Death Dis..

[120] Ma J., Shayiti F., Ma J., Wei M., Hua T., Zhang R., Su J., Chen P. (2021). Tumor-associated macrophage-derived CCL5 promotes chemotherapy resistance and metastasis in prostatic cancer. Cell Biol. Int..

[121] Ma G., Huang H., Li M., Li L., Kong P., Zhu Y., Xia T., Wang S. (2018). Plasma CCL5 promotes EMT-medicated epirubicin-resistance in locally advanced breast cancer. Cancer Biomarkers.

[122] Acharyya S., Oskarsson T., Vanharanta S., Malladi S., Kim J., Morris P.G., Manova-Todorova K., Leversha M., Hogg N., Seshan V.E. (2012). A CXCL1 Paracrine Network Links Cancer Chemoresistance and Metastasis. Cell.

[123] Wang N., Liu W., Zheng Y., Wang S., Yang B., Li M., Song J., Zhang F., Zhang X., Wang Q. (2018). CXCL1 derived from tumor-associated macrophages promotes breast cancer metastasis via activating NF-κB/SOX4 signaling. Cell Death Dis..

[124] Miyake M., Hori S., Morizawa Y., Tatsumi Y., Nakai Y., Anai S., Torimoto K., Aoki K., Tanaka N., Shimada K. (2016). CXCL1-Mediated Interaction of Cancer Cells with Tumor-Associated Macrophages and Cancer-Associated Fibroblasts Promotes Tumor Progression in Human Bladder Cancer. Neoplasia.

[125] Ullah T.R. (2019). The role of CXCR4 in multiple myeloma: Cells’ journey from bone marrow to beyond. J. Bone Oncol..

[126] Bao Y., Wang Z., Liu B., Lu X., Xiong Y., Shi J., Li P., Chen J., Zhang Z., Chen M. (2019). A feed-forward loop between nuclear translocation of CXCR4 and HIF-1α promotes renal cell carcinoma metastasis. Oncogene.

[127] Devignes C.S., Aslan Y., Brenot A., Devillers A., Schepers K., Fabre S., Chou J., Casbon A.J., Werb Z., Provot S. (2018). HIF signaling in osteoblast-lineage cells promotes systemic breast cancer growth and metastasis in mice. Proc. Natl. Acad. Sci. USA.

[128] Lu J., Zhong H., Chu T., Zhang X., Li R., Sun J., Zhong R., Yang Y., Alam A.S., Lou Y. (2019). Role of anlotinib-induced CCL2 decrease in anti-angiogenesis and response prediction for nonsmall cell lung cancer therapy. Eur. Respir. J..

[129] Hefler L., Tempfer C., Heinze G., Mayerhofer K., Breitenecker G., Leodolter S., Reinthaller A., Kainz C. (1999). Monocyte chemoattractant protein-1 serum levels in ovarian cancer patients. Br. J. Cancer.

[130] Lu X., Qian C.N., Mu Y.G., Li N.W., Li S., Zhang H.B., Li S.-W., Wang F.-L., Guo X., Xiang Y.-Q. (2011). Serum CCL2 and serum TNF-α–Two new biomarkers predict bone invasion, post-treatment distant metastasis and poor overall survival in nasopharyngeal carcinoma. Eur. J. Cancer.

[131] Monti P., Leone B.E., Marchesi F., Balzano G., Zerbi A., Scaltrini F., Pasquali C., Calori G., Pessi F., Sperti C. (2003). The CC chemokine MCP-1/CCL2 in pancreatic cancer progression: Regulation of expression and potential mechanisms of antimalignant activity. Cancer Res..

[132] Cai D., Xu Y., Ding R., Qiu K., Zhang R., Wang H., Huang L., Xie X., Yan H., Deng Y. (2020). Extensive serum biomarker analysis in patients with non-small-cell lung carcinoma. Cytokine.

[133] Sadeghi M., Lahdou I., Oweira H., Daniel V., Terness P., Schmidt J., Weiss K.-H., Longerich T., Schemmer P., Opelz G. (2015). Serum levels of chemokines CCL4 and CCL5 in cirrhotic patients indicate the presence of hepatocellular carcinoma. Br. J. Cancer.

[134] Zazo S., González-Alonso P., Martín-Aparicio E., Chamizo C., Luque M., Sanz-Álvarez M., Minguez P., Gomez-Lopez G., Cristobal I., Carames C. (2020). Autocrine CCL5 Effect Mediates Trastuzumab Resistance by ERK Pathway Activation in HER2-Positive Breast Cancer. Mol. Cancer Ther..

[135] Esteban E., Exposito F., Crespo G., Lambea J., Pinto A., Puente J., Arranz J.A., Redrado M., Rodriguez-Antona C., de Andrea C. (2021). Circulating Levels of the Interferon-γ-Regulated Chemokines CXCL10/CXCL11, IL-6 and HGF Predict Outcome in Metastatic Renal Cell Carcinoma Patients Treated with Antiangiogenic Therapy. Cancers.

[136] Chang C., Wang M., Bi X., Fan Z., Feng D., Cai H.Q., Zhang Y., Xu X., Cai Y., Qi J. (2021). Elevated serum eotaxin and IP-10 levels as potential biomarkers for the detection of esophageal squamous cell carcinoma. J. Clin. Lab. Anal..

[137] Koç Ü., Çetinkaya E., Bostanci E.B., Kemık A.S., Tez M., Gömceli İ., Akoglu M. (2013). Diagnostic Significance of Serum Eotaxin-1 Level in Gastric Cancer Patients. Dis. Markers.

[138] Wang J., Qin Y., Zhu G., Huang D., Wei M., Li G., She L., Zhang D., Wang G., Chen X. (2019). High serum CCL18 predicts a poor prognosis in patients with laryngeal squamous cell carcinoma. J. Cancer.

[139] Qin Y., Wang J., Zhu G., Li G., Tan H., Chen C., Pi L., She L., Chen X., Wei M. (2019). CCL18 promotes the metastasis of squamous cell carcinoma of the head and neck through MTDH-NF-κB signalling pathway. J. Cell Mol. Med..

[140] Sun J.H., Fan N., Zhang Y. (2016). Correlation between serum level of chemokine (C-C motif) ligand 18 and poor prognosis in breast cancer. Genet. Mol. Res..

[141] Plönes T., Krohn A., Burger M., Veelken H., Passlick B., Müller-Quernheim J., Zissel G. (2012). Serum Level of CC-Chemokine Ligand 18 Is Increased in Patients with Non-Small-Cell Lung Cancer and Correlates with Survival Time in Adenocarcinomas. PLoS ONE.

[142] Meng F., Li W., Li C., Gao Z., Guo K., Song S. (2015). CCL18 promotes epithelial-mesenchymal transition, invasion and migration of pancreatic cancer cells in pancreatic ductal adenocarcinoma. Int. J. Oncol..

[143] Hafidi M., Buelna-Chontal M., Sánchez-Muñoz F., Carbó R. (2019). Adipogenesis: A Necessary but Harmful Strategy. Int. J. Mol. Sci..

[144] Olbryt M., Habryka A., Student S., Jarząb M., Tyszkiewicz T., Lisowska K.M. (2014). Global Gene Expression Profiling in Three Tumor Cell Lines Subjected to Experimental Cycling and Chronic Hypoxia. PLoS ONE.

[145] Korbecki J., Simińska D., Gąssowska-Dobrowolska M., Listos J., Gutowska I., Chlubek D., Baranowska-Bosiacka I. (2021). Chronic and Cycling Hypoxia: Drivers of Cancer Chronic Inflammation through HIF-1 and NF-κB Activation: A Review of the Molecular Mechanisms. Int. J. Mol. Sci..

[146] Sun C., Li X., Guo E., Li N., Zhou B., Lu H., Huang J., Xia M., Shan W., Wang B. (2020). MCP-1/CCR-2 axis in adipocytes and cancer cell respectively facilitates ovarian cancer peritoneal metastasis. Oncogene.

[147] Rutkowski J.M., Davis K.E., Scherer P.E. (2009). Mechanisms of obesity and related pathologies: The macro- and microcirculation of adipose tissue. FEBS J..

[148] Halberg N., Khan T., Trujillo M.E., Wernstedt-Asterholm I., Attie A.D., Sherwani S., Wang Z.V., Landskroner-Eiger S., Dineen S., Magalang U.J. (2009). Hypoxia-Inducible Factor 1α Induces Fibrosis and Insulin Resistance in White Adipose Tissue. Mol. Cell Biol..

[149] Khan T., Muise E.S., Iyengar P., Wang Z.V., Chandalia M., Abate N., Zhang B.B., Bonaldo P., Chua S., Scherer P.E. (2009). Metabolic Dysregulation and Adipose Tissue Fibrosis: Role of Collagen VI. Mol. Cell Biol..

[150] Sun K., Tordjman J., Clément K., Scherer P.E. (2013). Fibrosis and Adipose Tissue Dysfunction. Cell Metab..

[151] Li X.P., Yang X.Y., Biskup E., Zhou J., Li H.L., Wu Y.F., Chen M.L., Xu F. (2015). Co-expression of CXCL8 and HIF-1α is associated with metastasis and poor prognosis in hepatocellular carcinoma. Oncotarget.

[152] Pavlides S., Whitaker-Menezes D., Castello-Cros R., Flomenberg N., Witkiewicz A.K., Frank P.G., Casimiro M.C., Wang C., Fortina P., Addya S. (2009). The reverse Warburg effect: Aerobic glycolysis in cancer associated fibroblasts and the tumor stroma. Cell Cycle.

[153] Migneco G., Whitaker-Menezes D., Chiavarina B., Castello-Cros R., Pavlides S., Pestell R.G., Fatatis A., Flomenberg N., Tsirigos A., Howell A. (2010). Glycolytic cancer associated fibroblasts promote breast cancer tumor growth, without a measurable increase in angiogenesis: Evidence for stromal-epithelial metabolic coupling. Cell Cycle.

[154] Castoldi A., Naffah de Souza C., Câmara N.O.S., Moraes-Vieira P.M. (2016). The Macrophage Switch in Obesity Development. Front. Immunol..

[155] Carlsen H., Haugen F., Zadelaar S., Kleemann R., Kooistra T., Drevon C.A., Blomhoff R. (2009). Diet-induced obesity increases NF-κB signaling in reporter mice. Genes. Nutr..

[156] Jin B.R., Kim H.J., Sim S.A., Lee M., An H.J. (2021). Anti-Obesity Drug Orlistat Alleviates Western-Diet-Driven Colitis-Associated Colon Cancer via Inhibition of STAT3 and NF-κB-Mediated Signaling. Cells.

[157] Široká M., Franco C., Guľašová Z., Hertelyová Z., Tomečková V., Rodella L., Rezzani R. (2020). Nuclear factor-kB and nitric oxide synthases in red blood cells: Good or bad in obesity? A preliminary study. Eur. J. Histochem..

[158] NF-kB Target Genes. https://www.bu.edu/nf-kb/gene-resources/target-genes/.

[159] Richmond A. (2002). NF-κB, chemokine gene transcription and tumour growth. Nat. Rev. Immunol..

[160] Hill A.A., Anderson-Baucum E.K., Kennedy A.J., Webb C.D., Yull F.E., Hasty A.H. (2015). Activation of NF-κB drives the enhanced survival of adipose tissue macrophages in an obesogenic environment. Mol. Metab..

[161] Camargo R., Riccardi D., Ribeiro H., Carnevali L., de Matos-Neto E., Enjiu L., Neves R.X., Correia Lima J.D.C., Figueredo R.G., Martins De Alcantara P.S. (2015). NF-κBp65 and Expression of Its Pro-Inflammatory Target Genes Are Upregulated in the Subcutaneous Adipose Tissue of Cachectic Cancer Patients. Nutrients.

[162] Zhang Y., Daquinag A.C., Amaya-Manzanares F., Sirin O., Tseng C., Kolonin M.G. (2012). Stromal Progenitor Cells from Endogenous Adipose Tissue Contribute to Pericytes and Adipocytes That Populate the Tumor Microenvironment. Cancer Res..

[163] Rosen E.D., Spiegelman B.M. (2014). What We Talk About When We Talk About Fat. Cell.

[164] Tseng C., Kolonin M.G. (2016). Proteolytic Isoforms of SPARC Induce Adipose Stromal Cell Mobilization in Obesity. Stem Cells.

[165] Zhang T., Tseng C., Zhang Y., Sirin O., Corn P.G., Li-Ning-Tapia E.M., Troncoso P., Davis J., Pettaway C., Ward J. (2016). CXCL1 mediates obesity-associated adipose stromal cell trafficking and function in the tumour microenvironment. Nat. Commun..

[166] Ma X., Liu J., Yang X., Fang K., Zheng P., Liang X., Liu J. (2020). Mesenchymal stem cells maintain the stemness of colon cancer stem cells via interleukin-8/mitogen-activated protein kinase signaling pathway. Exp. Biol. Med..

[167] Jotzu C., Alt E., Welte G., Li J., Hennessy B.T., Devarajan E., Krishnappa S., Pinilla S., Droll L., Song Y.H. (2011). Adipose tissue derived stem cells differentiate into carcinoma-associated fibroblast-like cells under the influence of tumor derived factors. Cell Oncol..

[168] Muehlberg F.L., Song Y.H., Krohn A., Pinilla S.P., Droll L.H., Leng X., Seidenstiker M., Ricke J., Altman A.M., Devarajan E. (2009). Tissue-resident stem cells promote breast cancer growth and metastasis. Carcinogenesis.

[169] Rowan B.G., Gimble J.M., Sheng M., Anbalagan M., Jones R.K., Frazier T.P., Asher M., Lacayo E.A., Friedlander P.L., Kutenr R. (2014). Human Adipose Tissue-Derived Stromal/Stem Cells Promote Migration and Early Metastasis of Triple Negative Breast Cancer Xenografts. PLoS ONE.

[170] Arendt L.M., McCready J., Keller P.J., Baker D.D., Naber S.P., Seewaldt V., Kuperwasser C. (2013). Obesity Promotes Breast Cancer by CCL2-Mediated Macrophage Recruitment and Angiogenesis. Cancer Res..

[171] Yao H., He S. (2021). Multi-faceted role of cancer-associated adipocytes in the tumor microenvironment (Review). Mol. Med. Rep..

[172] Wu Q., Li B., Li Z., Li J., Sun S., Sun S. (2019). Cancer-associated adipocytes: Key players in breast cancer progression. J. Hematol. Oncol..

[173] Al-Khalaf H.H., Al-Harbi B., Al-Sayed A., Arafah M., Tulbah A., Jarman A., Al-Mohanna F., Aboussekhra A. (2019). Interleukin-8 Activates Breast Cancer-Associated Adipocytes and Promotes Their Angiogenesis- and Tumorigenesis-Promoting Effects. Mol. Cell Biol..

[174] Fujisaki K., Fujimoto H., Sangai T., Nagashima T., Sakakibara M., Shiina N., Kuroda M., Aoyagi Y., Miyazaki M. (2015). Cancer-mediated adipose reversion promotes cancer cell migration via IL-6 and MCP-1. Breast Cancer Res. Treat..

[175] Hariharan N., Ashcraft K.A., Svatek R.S., Livi C.B., Wilson D., Kaushik D., Leach R.J., Johnson-Pais T.L. (2018). Adipose Tissue-Secreted Factors Alter Bladder Cancer Cell Migration. J. Obes..

[176] Sheng X., Tucci J., Parmentier J.H., Ji L., Behan J.W., Heisterkamp N., Mittelman S.D. (2016). Adipocytes cause leukemia cell resistance to daunorubicin via oxidative stress response. Oncotarget.

[177] Behan J.W., Yun J.P., Proektor M.P., Ehsanipour E.A., Arutyunyan A., Moses A.S., Avramis V.I., Louie S.G., Butturini A., Heisterkamp N. (2009). Adipocytes Impair Leukemia Treatment in Mice. Cancer Res..

[178] Bougaret L., Delort L., Billard H., Le Huede C., Boby C., De la Foye A., Rossary A., Mojallal A., Damour O., Auxenfans C. (2018). Adipocyte/breast cancer cell crosstalk in obesity interferes with the anti-proliferative efficacy of tamoxifen. PLoS ONE.

[179] Rahman S.M., Campbell J.M., Coates R.N., Render K.M., Byrne C.E., Martin E.C., Melvin A.T. (2020). Evaluation of intercellular communication between breast cancer cells and adipose-derived stem cells via passive diffusion in a two-layer microfluidic device. Lab. Chip.

[180] Li D., Ji H., Niu X., Yin L., Wang Y., Gu Y., Wang J., Zhou X., Zhang H., Zhang Q. (2020). Tumor-associated macrophages secrete CC-chemokine ligand 2 and induce tamoxifen resistance by activating PI3K/Akt/mTOR in breast cancer. Cancer Sci..

[181] Xu W., Wei Q., Han M., Zhou B., Wang H., Zhang J., Wang Q., Sun J., Feng L., Wang S. (2018). CCL2-SQSTM1 positive feedback loop suppresses autophagy to promote chemoresistance in gastric cancer. Int. J. Biol. Sci..

[182] Qian Y., Ding P., Xu J., Nie X., Lu B. (2022). CCL2 activates AKT signaling to promote glycolysis and chemoresistance in glioma cells. Cell Biol. Int..

[183] Wang T., Zhan Q., Peng X., Qiu Z., Zhao T. (2018). CCL2 influences the sensitivity of lung cancer A549 cells to docetaxel. Oncol. Lett..

[184] Yang Y.I., Wang Y.Y., Ahn J.H., Kim B.H., Choi J.H. (2022). CCL2 overexpression is associated with paclitaxel resistance in ovarian cancer cells via autocrine signaling and macrophage recruitment. Biomed. Pharmacother..

[185] Yan J., Gao Y., Lin S., Li Y., Shi L., Kan Q. (2022). EGR1-CCL2 Feedback Loop Maintains Epithelial-Mesenchymal Transition of Cisplatin-Resistant Gastric Cancer Cells and Promotes Tumor Angiogenesis. Dig. Dis. Sci..

[186] Kato T., Fujita Y., Nakane K., Mizutani K., Terazawa R., Ehara H., Kanimoto Y., Kojima T., Nozawa Y., Deguchi T. (2013). CCR1/CCL5 interaction promotes invasion of taxane-resistant PC3 prostate cancer cells by increasing secretion of MMPs 2/9 and by activating ERK and Rac signaling. Cytokine.

[187] Zhang X.N., Yang K.D., Chen C., He Z.C., Wang Q.H., Feng H., Lv S.Q., Wang Y., Mao M., Liu Q. (2021). Pericytes augment glioblastoma cell resistance to temozolomide through CCL5-CCR5 paracrine signaling. Cell Res..

[188] Yi E.H., Lee C.S., Lee J.K., Lee Y.J., Shin M.K., Cho C.H., Kang K.W., Lee J.W., Han W., Noh D.Y. (2013). STAT3-RANTES Autocrine Signaling Is Essential for Tamoxifen Resistance in Human Breast Cancer Cells. Mol. Cancer Res..

[189] Zhou B., Sun C., Li N., Shan W., Lu H., Guo L., Guo E., Xia M., Weng D., Meng L. (2016). Cisplatin-induced CCL5 secretion from CAFs promotes cisplatin-resistance in ovarian cancer via regulation of the STAT3 and PI3K/Akt signaling pathways. Int. J. Oncol..

[190] Gyanchandani R., Ortega Alves M.V., Myers J.N., Kim S. (2013). A Proangiogenic Signature Is Revealed in FGF-Mediated Bevacizumab-Resistant Head and Neck Squamous Cell Carcinoma. Mol. Cancer Res..

[191] Ploenes T., Scholtes B., Krohn A., Burger M., Passlick B., Müller-Quernheim J., Zissel G. (2013). CC-Chemokine Ligand 18 Induces Epithelial to Mesenchymal Transition in Lung Cancer A549 Cells and Elevates the Invasive Potential. PLoS ONE.

[192] Wang S., Lou N., Luo R., Hao X., Liu Y., Wang L., Shi Y., Han X. (2022). Role of chemokine-mediated angiogenesis in resistance towards crizotinib and its reversal by anlotinib in EML4-ALK positive NSCLC. J. Transl. Med..

[193] Fan T., Li S., Xiao C., Tian H., Zheng Y., Liu Y., Li C., He J. (2022). CCL20 promotes lung adenocarcinoma progression by driving epithelial-mesenchymal transition. Int. J. Biol. Sci..

[194] Geismann C., Grohmann F., Dreher A., Häsler R., Rosenstiel P., Legler K., Hauser C., Egberts J.H., Sipos B., Schreiber S. (2017). Role of CCL20 mediated immune cell recruitment in NF-κB mediated TRAIL resistance of pancreatic cancer. Biochim. Biophys. Acta - Mol. Cell Res..

[195] Chen M., Su J., Feng C., Liu Y., Zhao L., Tian Y. (2021). Chemokine CCL20 promotes the paclitaxel resistance of CD44 + CD117 + cells via the Notch1 signaling pathway in ovarian cancer. Mol. Med. Rep..

[196] Su S., Sun X., Zhang Q., Zhang Z., Chen J. (2019). CCL20 Promotes Ovarian Cancer Chemotherapy Resistance by Regulating ABCB1 Expression. Cell Struct. Funct..

[197] Wang D., Yang L., Yu W., Wu Q., Lian J., Li F., Liu S., Li A., He Z., Liu J. (2019). Colorectal cancer cell-derived CCL20 recruits regulatory T cells to promote chemoresistance via FOXO1/CEBPB/NF-κB signaling. J. Immunother. Cancer.

[198] Park Y.L., Kim H.P., Ock C.Y., Min D.W., Kang J.K., Lim Y.J., Song S.H., Han S.W., Kim T.Y. (2022). EMT-mediated regulation of CXCL1/5 for resistance to anti-EGFR therapy in colorectal cancer. Oncogene.

[199] Wang C., Li A., Yang S., Qiao R., Zhu X., Zhang J. (2018). CXCL5 promotes mitomycin C resistance in non-muscle invasive bladder cancer by activating EMT and NF-κB pathway. Biochem. Biophys. Res. Commun..

[200] Giuliano S., Dufies M., Ndiaye P.D., Viotti J., Borchiellini D., Parola J., Vial V., Cormerais Y., Ohanna M., Imbert V. (2019). Resistance to lysosomotropic drugs used to treat kidney and breast cancers involves autophagy and inflammation and converges in inducing CXCL5. Theranostics.

[201] Simoncello F., Piperno G.M., Caronni N., Amadio R., Cappelletto A., Canarutto G., Piazza S., Bicciato S., Benvenuti F. (2022). CXCL5-mediated accumulation of mature neutrophils in lung cancer tissues impairs the differentiation program of anticancer CD8 T cells and limits the efficacy of checkpoint inhibitors. Oncoimmunology.

[202] Stronach E.A., Cunnea P., Turner C., Guney T., Aiyappa R., Jeyapalan S., deSousa C.H., Browne A., Magdy N., Studd J.B. (2015). The role of interleukin-8 (IL-8) and IL-8 receptors in platinum response in high grade serous ovarian carcinoma. Oncotarget.

[203] Liu Y.N., Chang T.H., Tsai M., Wu S.G., Tsai T.H., Chen H.Y., Yu S.L., Yang J.C.H., Shih J.Y. (2015). IL-8 confers resistance to EGFR inhibitors by inducing stem cell properties in lung cancer. Oncotarget.

[204] Zhang H., Yu Q.L., Meng L., Huang H., Liu H., Zhang N., Liu N., Yang J., Zhang Y.Z., Huang Q. (2020). TAZ-regulated expression of IL-8 is involved in chemoresistance of hepatocellular carcinoma cells. Arch. Biochem. Biophys..

[205] Imafuji H., Matsuo Y., Ueda G., Omi K., Hayashi Y., Saito K., Tsuboi K., Morimoto M., Koide S., Ogawa R. (2019). Acquisition of gemcitabine resistance enhances angiogenesis via upregulation of IL-8 production in pancreatic cancer. Oncol. Rep..

[206] Song Y., Baba T., Li Y.Y., Furukawa K., Tanabe Y., Matsugo S., Sasaki S., Mukaida N. (2015). Gemcitabine-induced CXCL8 expression counteracts its actions by inducing tumor neovascularization. Biochem. Biophys. Res. Commun..

[207] Wu S., Saxena S., Varney M.L., Singh R.K. (2018). CXCR1/2 Chemokine Network Regulates Melanoma Resistance to Chemotherapies Mediated by NF-κB. Curr. Mol. Med..

[208] Huang T.H., Mokgautsi N., Huang Y.J., Wu A.T.H., Huang H.S. (2021). Comprehensive Omics Analysis of a Novel Small-Molecule Inhibitor of Chemoresistant Oncogenic Signatures in Colorectal Cancer Cell with Antitumor Effects. Cells.

[209] Gelfo V., Rodia M.T., Pucci M., Dall’Ora M., Santi S., Solmi R., Roth L., Lindzen M., Bonafe M., Bertotti A. (2016). A module of inflammatory cytokines defines resistance of colorectal cancer to EGFR inhibitors. Oncotarget.

[210] Du J., He Y., Li P., Wu W., Chen Y., Ruan H. (2018). IL-8 regulates the doxorubicin resistance of colorectal cancer cells via modulation of multidrug resistance 1 (MDR1). Cancer Chemother. Pharmacol..

[211] Britschgi A., Radimerski T., Bentires-Alj M. (2013). Targeting PI3K, HER2 and the IL-8/JAK2 axis in metastatic breast cancer: Which combination makes the whole greater than the sum of its parts?. Drug Resist. Updat..

[212] Xia L., Yang F., Wu X., Li S., Kan C., Zheng H., Wang S. (2021). SHP2 inhibition enhances the anticancer effect of Osimertinib in EGFR T790M mutant lung adenocarcinoma by blocking CXCL8 loop mediated stemness. Cancer Cell Int..

[213] Ahmed S., Mohamed H.T., El-Husseiny N., El Mahdy M.M., Safwat G., Diab A.A., El-Sherif A.A., El-Shinawi M., Mohamed M.M. (2021). IL-8 secreted by tumor associated macrophages contribute to lapatinib resistance in HER2-positive locally advanced breast cancer via activation of Src/STAT3/ERK1/2-mediated EGFR signaling. Biochim. Biophys. Acta Mol. Cell Res..

[214] Kikuchi H., Maishi N., Annan D.A., Alam M.T., Dawood R.I.H., Sato M., Morimoto M., Takeda R., Ishizuka K., Matsumoto R. (2020). Chemotherapy-Induced IL8 Upregulates MDR1/ABCB1 in Tumor Blood Vessels and Results in Unfavorable Outcome. Cancer Res..

[215] Milosevic V., Kopecka J., Salaroglio I.C., Libener R., Napoli F., Izzo S., Orecchia S., Ananthanarayanan P., Bironzo P., Grosso F. (2020). Wnt/IL-1β/IL-8 autocrine circuitries control chemoresistance in mesothelioma initiating cells by inducing ABCB5. Int. J. Cancer.

[216] Zhai J., Shen J., Xie G., Wu J., He M., Gao L., Zhang Y., Yao X., Shen L. (2019). Cancer-associated fibroblasts-derived IL-8 mediates resistance to cisplatin in human gastric cancer. Cancer Lett..

[217] Wilson C., Purcell C., Seaton A., Oladipo O., Maxwell P.J., O’Sullivan J.M., Wilson R.H., Johnston P.G., Waugh D.J.J. (2008). Chemotherapy-Induced CXC-Chemokine/CXC-Chemokine Receptor Signaling in Metastatic Prostate Cancer Cells Confers Resistance to Oxaliplatin through Potentiation of Nuclear Factor-κB Transcription and Evasion of Apoptosis. J. Pharmacol. Exp. Ther..

[218] Wu X., Sun A., Yu W., Hong C., Liu Z. (2020). CXCL10 mediates breast cancer tamoxifen resistance and promotes estrogen-dependent and independent proliferation. Mol. Cell Endocrinol..

[219] Delitto D., Perez C., Han S., Gonzalo D.H., Pham K., Knowlton A.E., Graves C.L., Behrns K.E., Moldawer L.L., Thomas R.M. (2015). Downstream mediators of the intratumoral interferon response suppress antitumor immunity, induce gemcitabine resistance and associate with poor survival in human pancreatic cancer. Cancer Immunol. Immunother..

[220] Gómez A.M., Martínez C., González M., Luque A., Melen G.J., Martínez J., Hortelano S., Lassaletta A., Madero L., Ramirez M. (2015). Chemokines and relapses in childhood acute lymphoblastic leukemia: A role in migration and in resistance to antileukemic drugs. Blood Cells, Mol. Dis..

[221] Chen D.L., Sheng H., Zhang D.S., Jin Y., Zhao B.T., Chen N., Song K., Xu R.H. (2021). The circular RNA circDLG1 promotes gastric cancer progression and anti-PD-1 resistance through the regulation of CXCL12 by sponging miR-141-3p. Mol. Cancer.

[222] Tang H., Long Q., Zhuang K., Han K., Zhang X., Guo H., Lu X. (2021). Retinoblastoma tumor suppressor gene 1 enhances 5-Fluorouracil chemosensitivity through SDF-1/CXCR4 axis by regulating autophagy in gastric cancer. Pathol. Res. Pract..

[223] Singh S., Srivastava S.K., Bhardwaj A., Owen L.B., Singh A.P. (2010). CXCL12–CXCR4 signalling axis confers gemcitabine resistance to pancreatic cancer cells: A novel target for therapy. Br. J. Cancer.

[224] Wei L., Ye H., Li G., Lu Y., Zhou Q., Zheng S., Lin Q., Liu Y., Li Z., Chen R. (2018). Cancer-associated fibroblasts promote progression and gemcitabine resistance via the SDF-1/SATB-1 pathway in pancreatic cancer. Cell Death Dis..

[225] Zhang F., Cui J., Gao H., Yu H., Gao F., Chen J., Chen L. (2020). Cancer-associated fibroblasts induce epithelial-mesenchymal transition and cisplatin resistance in ovarian cancer via CXCL12/CXCR4 axis. Futur. Oncol..

[226] Agarwal P., Isringhausen S., Li H., Paterson A.J., He J., Gomariz Á., Nagasawa T., Nombela-Arieta C., Bhatia R. (2019). Mesenchymal Niche-Specific Expression of Cxcl12 Controls Quiescence of Treatment-Resistant Leukemia Stem Cells. Cell Stem Cell.

[227] Yu X., Shi W., Zhang Y., Wang X., Sun S., Song Z., Liu M., Zeng Q., Cui S., Qu X. (2017). CXCL12/CXCR4 axis induced miR-125b promotes invasion and confers 5-fluorouracil resistance through enhancing autophagy in colorectal cancer. Sci. Rep..

[228] Wang S., Chen C., Li J., Xu X., Chen W., Li F. (2020). The CXCL12/CXCR4 axis confers temozolomide resistance to human glioblastoma cells via up-regulation of FOXM1. J. Neurol. Sci..

[229] Onishi I., Nakagawa Y., Murayama T., Hidaka M., Yamamoto K., Abe-Suzuki S., Abe S., Kurata M., Kitagawa M. (2014). Expression of multidrug resistance 1 gene in association with CXCL12 in chronic myelogenous leukaemia. Pathology.

[230] Cena H., Calder P.C. (2020). Defining a healthy diet: Evidence for the role of contemporary dietary patterns in healthy and disease. Nutrients.

[231] Annett S., Moore G., Robson T. (2020). Obesity and Cancer Metastasis: Molecular and Translational Perspectives. Cancers.

[232] Hackman G.L., Collins M., Lu X., Lodi A., DiGiovanni J., Tiziani S. (2020). Predicting and Quantifying Antagonistic Effects of Natural Compounds Given with Chemotherapeutic Agents: Applications for High-Throughput Screening. Cancers.

[233] Huang Z., Shi Y., Bao P., Cai H., Hong Z., Ding D., Jackson J., Shu X.O., Dai Q. (2018). Associations of dietary intake and supplement use with post-therapy cognitive recovery in breast cancer survivors. Breast Cancer Res. Treat..

[234] Ambrosone C.B., Zirpoli G.R., Hutson A.D., McCann W.E., McCann S.E., Barlow W.E., Kelly K.M., Cannioto R., Sucheston-Campbell L.E., Hershman D.L. (2020). Dietary Supplement Use During Chemotherapy and Survival Outcomes of Patients With Breast Cancer Enrolled in a Cooperative Group Clinical Trial (SWOG S0221). J. Clin. Oncol..

